# *Streptococcus* spp. in oral cancer: host-microbe interactions, mechanistic insights, and diagnostic implications

**DOI:** 10.3389/fcimb.2025.1688701

**Published:** 2025-11-24

**Authors:** Srikrishna Kedlaya Herga, Adarsh Kudva, Suchitra Shenoy M., Thanvanthri Gururajan Vasudevan, Vijendra Prabhu, Raghavendra Upadhya, A. S. Bharath Prasad

**Affiliations:** 1 Department of Public Health Genomics, Manipal School of Life Sciences, Manipal Academy of Higher Education, Manipal, Karnataka, India; 2 Department of Oral and Maxillofacial Surgery, Manipal College of Dental Sciences, Manipal Academy of Higher Education, Manipal, Karnataka, India; 3 Department of Microbiology, Kasturba Medical College Mangalore, Manipal Academy of Higher Education, Mangalore, Karnataka, India; 4 Center for Cellular and Molecular Sciences, Shri Dharmasthala Manjunatheshwara University, Dharwad, Karnataka, India; 5 Photoceutics and Regeneration Laboratory, Department of Biotechnology, Manipal Institute of Technology, Manipal Academy of Higher Education, Manipal, Karnataka, India; 6 Center for Microfluidics, Biomarkers, Photoceutics and Sensors (μBioPS), Manipal Institute of Technology, Manipal Academy of Higher Education, Manipal, Karnataka, India; 7 Department of Biotherapeutics Research, Manipal Academy of Higher Education, Manipal, Karnataka, India

**Keywords:** biomarkers, cancer microbiome, host-bacteria crosstalk, inflammation, oral cancer, oral microbiota, streptococci

## Abstract

Cancers of the oral cavity, particularly the widely prevalent oral squamous cell carcinoma, are associated with microbial dynamics within the oral niche. Among them, oral *Streptococcus* sp. – once neglected as a commensal habitat of the oral cavity – is currently highlighted for its pivotal dual interplay in the progression and suppression of oral cancers. In this comprehensive review, we describe the association of these oral streptococcal species with oral cancer in detail – right from their abundance and depletion during the progression of the disease, mechanistic synergy involving factors such as the surface receptors playing an intricate role in biofilm and co-adhesion strategies, to the inflammatory interplay in cancerous cells, and metabolic reprogramming associated with oral cancer. We also highlight oncogenic and onco-mitigating oral streptococci as biomarkers, observing a complex microbial interaction regulating tumor initiation and development. This review serves as a novel direction to address streptococcal mediators in oral cancer by bridging research gaps in mechanistic evidence and proposing effective prospects that can address deeper exploration of streptococcal dualistic role in the tumor microenvironment to decipher effective theragnostic strategies to manage oral cancer.

## 1 Introduction

Diseases of the oral cavity are one of the most predominant and common diseases around the globe. They are a result of chronic and progressive conditions leading to infection in the tooth and mouth (Peres et al., 2019; Botelho et al., 2022). They encompass various clinical manifestations, from minor diseases such as periodontitis, gingivitis, and dental caries to complicated and systemic diseases, including Noma, oro-dental trauma, oral cancers, and cancerous ulcers. The 2022 Global Oral Health Status Report, published by the World Health Organization (WHO), estimates that about 3.5 billion people worldwide are diagnosed with oral diseases (World Health Organization, 2022). The microbiome of the oral cavity is the key influencing factor in oral health and diseases. The oral cavity harbors a multitude of microorganisms with a diverse composition of species (Lamont et al., 2018). Right after birth, microbes colonize the buccal cavity of the infant (Nuriel-Ohayon et al., 2016; Tuominen et al., 2019). The microflora of the mouth becomes more complicated and diverse with gradual aging, and it depends on the diet, lifestyle, and physiological conditions, such as the influence of saliva, digestive enzymes, pH, oxygen availability in different anatomical locations of the mouth, and infections (Kilian, 2018). Human oral microflora hosts about 700 different species of bacteria, of which about 250 different species have been isolated and characterized from the oral cavity (Deo and Deshmukh, 2019; Sedghi et al., 2021).

The human oral microflora is collectively compiled in the eHOMD, expanded Human Oral Microflora Database (expanded Human Oral Microbiome Database Version 3.1, n.d.). This database utilizes 16S rRNA gene sequencing as a phylogenetic and taxonomic marker to identify the diverse oral microflora in humans (Gevers et al., 2012). It is rich in curated information about various bacteria that inhabit the human mouth and the aerodigestive tract, including the pharynx, nasal passages, sinuses, and esophagus. According to version 3.1, there are 774 oral bacterial species, of which 58% have an official name, 16% are unnamed but cultured, and 26% are uncultivated phylotypes. eHOMD taxonomy offers a provisional naming scheme for currently unnamed taxa, based on the 16S rRNA gene sequence phylogeny, so that any strain, clone, and probe data from any laboratory can be directly linked to a stably named reference scheme (expanded Human Oral Microbiome Database Version 3.1, n.d.). Based on the entries in the database, *Streptococcus* and related families constitute one of the dominant occupants in the oral microbiota (Gao et al., 2018). Irrespective of eating patterns, habits, or infections, different species of streptococci ubiquitously inhabit the oral cavity. Indeed, a recent genomic catalog of cultivated human oral bacteria found *Streptococcus* to be the most dominant genus, representing nearly 58% (625 genomes) of the high-quality genomes assembled (Li et al., 2023a). Based on the anatomical distributions, *Streptococcus* sp. is found to be the most abundant species in mucosal tissue, comprising about 44% of the oral microflora of the hard palate, 65% of the oral mucosa, and 66% of keratinized gingiva. They also represent about 12% of the microflora of the tongue, 13% of supragingival plaque, 15% of subgingival plaque, and 23% and 15% of saliva, with and without rinsing, respectively (Caselli et al., 2020). Though prevalent, not all streptococci are commensal. Streptococcal pathogens are frequently reported in dysbiosis of the oral cavity (Sudhakara et al., 2018). Many oral streptococcal species such as *S. mutans* (Kojima et al., 2012; Mattos-Graner et al., 2014; Ito et al., 2019; Lemos et al., 2019; Latifi-Xhemajli et al., 2021; Zhang et al., 2022)*, S. intermedius* (Socransky et al., 1998; Haruta et al., 2008; Tran et al., 2008; Neumayr et al., 2010; Issa et al., 2020; Zhu et al., 2024)*, S. constellatus* (Rams et al., 2014; Faden and Mohmand, 2017; Kobo et al., 2017; Chrastek et al., 2020; Glover et al., 2020; Jiang et al., 2020; Vulisha et al., 2021; Xia et al., 2021; Rostami et al., 2022; Farias et al., 2023)*, S. oralis* (Ren et al., 2024, 2025), and *S. pyogenes* (Hung et al., 2024) are associated with infections, from mild systemic infections, periodontitis, and cavities to severe life-threatening infections such as abscesses in the brain, liver dysfunction, and endocarditis. Some key oral streptococci were even reported in Oral Squamous Cell Carcinoma (OSCC) patient samples (Senthil Kumar et al., 2024a; Sukmana et al., 2024). Nevertheless, few studies have specifically looked at their involvement in the development and progression of oral cancer.

**Figure 1:**
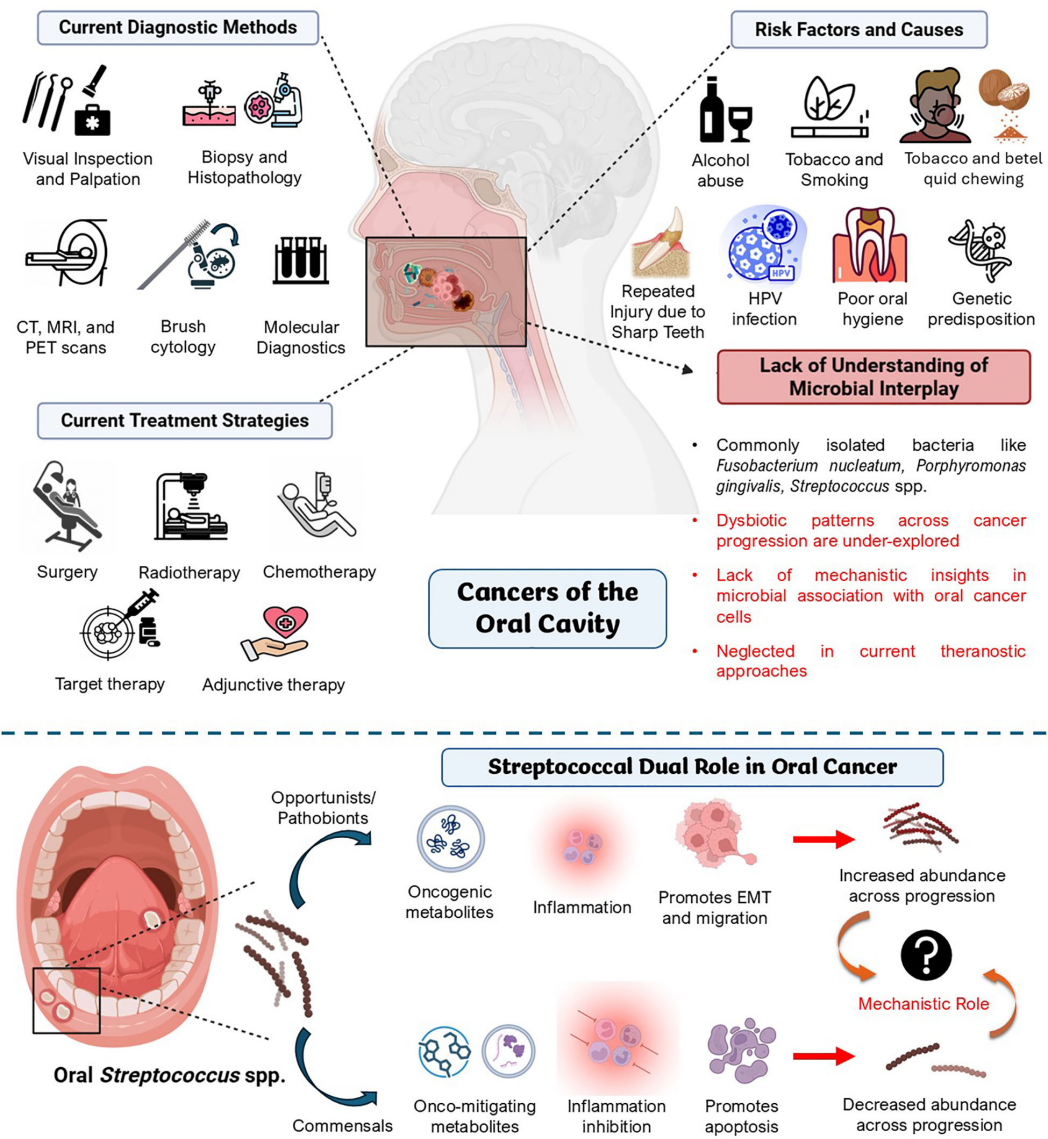
Current clinical overview of oral cancer – risk factors, diagnostic methods, and therapeutic approaches. There is an apparent lack of understanding of microbial interplay in oral cancer theragnostics. The figure also describes the research gap in understanding the mechanistic role of oral *Streptococcus* spp., the predominant species in the oral cavity, in oral cancer, which plays synergistic and antagonistic roles in oral cancer pathology. Created using BioRender.

Oral cancer is a significant systemic disorder of the oral cavity with the highest global health burden, given its prevalence as one of the most common head and neck malignancies reported worldwide (Tan et al., 2023). It is characterized by the growth and proliferation of cancerous tissues in and across the lips, tongue, cheeks, floor of the mouth, soft and hard palate, sinuses, and throat. About 90% of all reported cases of oral cancer are reported as OSCC (Coletta et al., 2024). The highest burden of OSCC is seen in South and Southeast Asian countries, with India being referred to as the “Oral Cancer Capital of the World” (Das and Motghare, 2021). According to the National Institute of Dental and Craniofacial Research (NIDCR), USA, the overall 5-year survival rate of subjects with oral cancer is approximately 68% which calls for effective diagnostic and therapeutic plans to manage it (National Institute of Dental and Craniofacial Research, 2025). Various risk factors account for its onset, the most common being tobacco chewing, smoking cigarettes, betel quid chewing, and even Human Papillomavirus (HPV) infection (Tan et al., 2023; Coletta et al., 2024; Ryntathiang et al., 2024). Though these risk factors take a considerable share, cases have been documented where subjects were diagnosed with oral cancer without being exposed to these risk factors (Saxena and Kumar, 2019). While diagnostic approaches consider these risk factors, the prevalence of microbial association, dysbiotic flora, and lifestyle changes is often left unnoticed, leading to late diagnoses, higher morbidity, and mortality rates (Gangane et al., 2024). Treatments, including surgical removal of tumors and cancerous nodes, radiation, chemo, and immune therapies, have been proven to treat oral cancer effectively, but, substantial clinical gap exists in the prevention and screening of ulcerative or pre-malignant stages (Tan et al., 2023). Considering the complexity of this disease in relation to the genetic, environmental, lifestyle, and microbial factors, there is an urge to discover and develop biomarkers for early diagnosis and targeted therapy (**Figure 1**) (Ryntathiang et al., 2024).

Given the inadequacies in current techniques to diagnose and treat oral cancer, such as limited sensitivity of current diagnostic markers, late-stage detection, lack of specificity in targeted treatments, and economic burden on patients, there is a growing interest in identifying various microbial signatures across oral cancer patients to assist early theragnostic interventions (Kleinstein et al., 2020; Yang et al., 2025). Among these, there have been reports of an abundance of some key streptococcal interplay with the tumor compared to non-cancerous conditions, with clinical research showing the increased presence of these pathogens in patients with oral cancer (Mäkinen et al., 2023; Senthil Kumar et al., 2024a, 2024b; Zhou et al., 2024). While current studies focus on unraveling the role of oral microbiota as potential co-morbidities in oral cancer, the exact ways in which some key *Streptococcus* spp. affect oral cancer progression are still not fully understood. This review aims to fill this gap by thoroughly analyzing the link between *Streptococcus* spp. and oral cancer. Unlike earlier reviews that cover the oral microbiome in general, we focus specifically on *Streptococcus* spp. and their association with cancers of the oral cavity. This review delves into its possible cancer-causing and cancer-preventive mechanisms, the role of *Streptococcus*-mediated inflammation, changes in the immune system, metabolic changes, and biofilm formation as potential associated factors of oral cancer. We also discuss how shifts in *Streptococcus*-related microbes could serve as diagnostic markers and treatment targets to offer a fresh perspective on their association with oral cancer. By summarizing recent research and pointing out research gaps, this review aims to shed light on complex interactions of *Streptococcus* and related species in oral cancer by synthesizing conceptual models for key metabolic pathways involved in this host-microbe interaction, which can be used to explore microbe-based biomarkers for developing theragnostic strategies in oral cancer, potentially paving way for new perceptions into microbial contributions to oral cancer pathogenesis and subsequent translational applications in diagnosis and treatment.

## 2 *Streptococcus* spp. implicated in oral cancer – microbiome and metagenomic studies

Oral streptococci are facultative anaerobic, Gram-positive bacteria. They are one of the earliest colonizers of the buccal cavity. After the child’s birth, many bacteria, including species of *Streptococcus*, colonize the oral cavity through breastfeeding, breathing, and personal contact (Smith et al., 1993; Li et al., 2023b). Oral *Streptococcus* spp. play a dual role in oral health and contribute to diseases. Commensal species such as *Streptococcus sanguinis*, *Streptococcus salivarius, Streptococcus mitis*, and *Streptococcus oralis* are primary colonizers of the mouth. They help protect oral health by competing with harmful pathogens, thus preventing pathogenic overgrowth (Abranches et al., 2018; Senthil Kumar et al., 2024b). However, some *Streptococcus* species are linked to oral diseases (Spatafora et al., 2024). *Streptococcus mutans* and *Streptococcus sobrinus* are known to cause cavities by producing acids that break down enamel (Abranches et al., 2018; Nomura et al., 2020; Korona-Glowniak et al., 2022; Spatafora et al., 2024). *Streptococcus anginosus* and *Streptococcus intermedius* are also connected to serious oral infections, such as abscesses, bacteremia, and sepsis (Neumayr et al., 2010; Issa et al., 2020; Jiang et al., 2020; Pilarczyk-Zurek et al., 2022; Furuholm et al., 2023; Reyes et al., 2023; Zhu et al., 2024). Recent studies also suggest that some *Streptococcus* species might play a role in oral cancer by causing chronic inflammation, modulating immune responses, altering the host metabolism, and promoting tumorigenesis (Chocolatewala et al., 2010; Karpiński, 2019; Yu et al., 2022; Senthil Kumar et al., 2024b, 2024a; Sukmana et al., 2024; Zhou et al., 2024).

### 2.1 Streptococcal isolates in oral cancer cases – what do they indicate?

**Figure 2:**
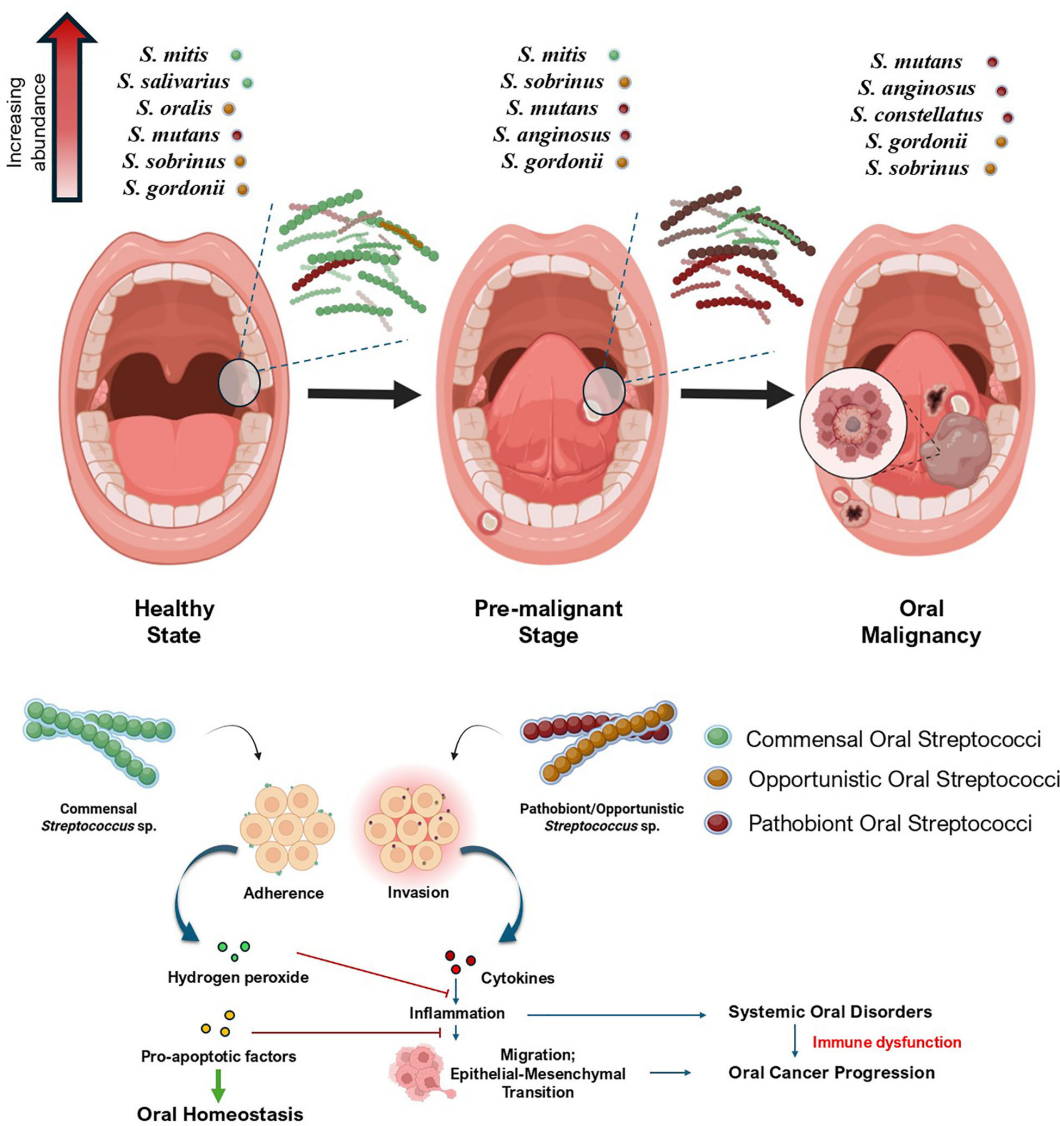
Association of oral streptococci during the progression of oral cancer. The figure shows the shift in oral streptococcal abundance across the transition from a healthy state to oral malignancy. The synergy of opportunistic and pathobiont streptococci that helps in systemic oral cancer progression and the antagonistic interplay of commensal species in regulating these events to maintain homeostasis has also been portrayed. Created using BioRender.

Recent clinical studies have explored how different *Streptococcus* spp. relate to oral cancer. These studies found notable differences in the types and quantities of specific *Streptococcus* spp. between oral cancer patients and healthy people during bacterial profiling of their oral microbiota. These studies hint that these bacteria might play a role in either the progression or prevention of oral cancer (**Figure 2****).**

#### 2.1.1 Streptococcal dysbiosis in oral cancer: insights into the commensal–pathogen duality

Several studies have implicated the dysbiotic trends of *Streptococcus* sp. in oral cancer tissues compared to non-cancerous oral tissues from subjects. Although presented below in a thematic sequence to emphasize key microbial patterns in oral cancer, the studies span different years and cohorts. In a prominent study by Tsai et al. (2022), which included a cohort of 82 individuals, it was found that *S. mutans* was more frequently observed in patients with OSCC (**Supplementary Figure S1a**). The researchers analyzed oral biofilms and discovered distinct microbial compositions between OSCC patients and healthy individuals. This highlighted the significant presence of *S. mutans* linked to the development of OSCC. Additional analysis using 16S rRNA PCR revealed that *S. mutans* was associated with even clinically advanced stages of OSCC and was found to worsen disease management in OSCC patients (Tsai et al., 2022). An earlier study by Torralba et al. (2021) showed essential differences in microbes between OSCC tumor tissues (TT, *n=*18) and nearby non-tumor tissues (NT, *n=*18) from the cohorts diagnosed with oral cancer. It was observed that *Streptococcus gordonii* was strongly linked to OSCC tissues. Notably, *S. gordonii* was found to co-exist alongside *Fusobacterium* species. Metagenomic analysis found that *S. gordonii* had higher levels of fibronectin-binding proteins CshA and CshB in TT. This is significant because *S. gordonii* is an early to mid-colonizer in oral biofilms, and its presence could be a valuable marker for diagnosing OSCC. Moreover, while *S. gordonii* was closely associated with OSCC, *S. anginosus* was more abundant in TT samples. Thus, there is a shift in microbial balance as OSCC progresses. The study also found that TT samples contained more well-known oral pathogens such as *Prevotella, Parvimonas micra*, *Fusobacterium*, and *S. anginosus* (**Supplementary Figure S1b**). These results highlight a significant change in the oral microbiome in OSCC as it progresses (Torralba et al., 2021). Zhou et al. (2024) reported that *S. mutans* are gradually involved in spatial shifting from non-tumorous adjacent tissues to OSCC tissues as the disease progresses (**Supplementary Figure S1c**). Their study analyzed saliva and swab samples of 17 healthy controls and 28 OSCC patients, as well as paired tumor tissues and adjacent non-tumor tissues from 24 OSCC patients. qPCR and fluorescence *in situ* hybridization (FISH) were used to investigate the relative abundance and spatial distribution of different *Streptococcus* spp. in OSCC patients (**Supplementary Figure S1d**). The findings indicate that *S. mutans*, compared to other *Streptococcus* spp. such as *S. sanguinis, S. parasanguinis, S. salivarius, S. mitis*, and *S. gordonii*, are involved more in spatial shifting to tumorous tissues, which was confirmed by FISH (**Supplementary Figure S1e**). This study also hinted at the expression of kynurenic acid as a biomarker because of metabolic reprogramming by *S. mutans* in the saliva of OSCC patients to determine the progression of OSCC (Zhou et al., 2024).

While the aforementioned studies reveal that certain species of *Streptococcus*, such as *S. mutans* and *S. angiosus*, are abundant in oral cancer tumor sites, several clinical and metagenomic studies indicate the inverse analogy of streptococcal abundance. In a recent study conducted by Yang et al. (2025), where mucosa-associated microbiomes in normal, precancerous, and OSCC lesions from a cohort of 51 patients were analyzed by 16S rRNA amplicon sequencing, it was evident that *Streptococcus* groups were decreased in OSCC cases with the increase in numbers of *Fusobacterium* and *Prevotella*, both of which are linked to oral cancer (**Supplementary Figure S2a**). However, it is notable that in normal and pre-cancerous conditions, the oral microbiota harbored more *Streptococcus* spp. Using linear discriminant analysis and Spearman correlation, they found that the decrease in *Streptococcus*, along with an increase in the bacteria linked to oral cancer, marking a key change in the microbial population towards a cancerous state (Yang et al., 2025). Banavar et al. (2021) analyzed meta-transcriptomic data from saliva samples (n = 433) collected from oral pre-malignant disorders, OC patients (n = 71) and normal controls (n = 171). They also found that 39 of the 41 differentially active species from the *Streptococcus* genus were downregulated along with other commensal bacterial groups such as *Hemophilus* and *Actinomyces* in oral cancer cases, with the reason unstated. However, it is to be noted that this study also suggested an increase in numbers of *Porphyromonas, Fusobacterium*, and *Prevotella* in the diseased state (Banavar et al., 2021). Interestingly, this phenomenon had been reported earlier by Yang et al. (2018), where the relative abundance of commensal *Streptococcus* spp. like *S. mitis* had significantly reduced in oral cancer cases, along with other commensals such as *Hemophilus parainfluenzae, Porphyromonas pasteri, Veillonella parvula*, and *Actinomyces odontolyticus* (**Supplementary Figure S2b**). This study involved bacterial metagenomic profiling across the oral rinse samples from 51 healthy individuals and 197 OSCC patients by the 16S rRNA V3V4 amplicon sequencing method (Yang et al., 2018). Mok et al. (2017) had also previously reported a similar trend, where it was observed that *Streptococcus* spp. was found to be relatively higher in normal, Oral Potentially Malignant Disorder (OPMD) cases, and oral cancer cases. However, the abundance of *Streptococcus* species decreased from normal to OPMD and oral cancer conditions (**Supplementary Figure S2c**) (Mok et al., 2017). Together, these studies corroborate that a reduction in commensal *Streptococcus* spp. is evident across oral cancer progression.

#### 2.1.2 Species-specific implications for *Streptococcus* in oral cancer

A complex relationship exists between *Streptococcus* spp. and oral cancer conditions, as supported by various clinical studies and analyses of oral microbiota to date. Different groups and species of *Streptococcus* have varying effects, promoting oral cancer by pro-oncogenic influences or providing protective benefits, demonstrating anti-oncogenic properties. The pernicious effects may arise from some pathobiont *Streptococcus* spp., which can promote chronic inflammation and oxidative damage *in situ*. This potentially supports tumorigenesis and the development of the tumor microenvironment. On the other hand, certain commensal *Streptococcus* spp. like *S. mitis* help keep the buccal cavity healthy by counteracting cancer progression and causing cellular damage to cancerous cells, leading to cell death (Inui et al., 2025). The interaction between these microbial groups and the tumor environment shows the intricacies of microbial dynamics in OSCC. Understanding these microbial dynamics is crucial for identifying microbial markers and developing targeted therapeutics for oral cancers. The association of different streptococcal groups and species with oral cancer cases, along with their implications, is listed in detail in **Table 1**.

**Table 1:** *Streptococcus* implications with oral cancer cases. (OSCC – Oral Squamous Cell Carcinoma).

Species	Study type	Abundance findings	Implications	References
*Streptococcus mutans*	Clinical, lab-based, and preclinical	Increased presence in OSCC tissues	Promotes tumor progression by increasing IL-6 production and immunometabolism modulation	(Tsai et al., 2022; Zhou et al., 2024)
*Streptococcus anginosus*	Clinical; laboratory-based findings	Detected in cancer samples from oral and pharyngeal regions	Induces chronic inflammation through proinflammatory cytokines; metabolizes ethanol to acetaldehyde, contributing to carcinogenesis	(Morita et al., 2003; Sasaki et al., 2005; Senthil Kumar et al., 2024a)
*Streptococcus mitis*	Clinical	Elevated levels in the saliva of OSCC patients; decrease as the disease progresses	Potential early tumor markers may inhibit tumor cell proliferation through hydrogen peroxide production	(Zhao et al., 2017; Yang et al., 2018; Baraniya et al., 2020)
*Streptococcus gordonii*	Clinical	Associated with tumor sites in OSCC patients	Potential role in tumor development; mechanisms not fully elucidated	(Torralba et al., 2021)
*Streptococcus parasanguinis*	Clinical	Altered abundance in salivary microbiota of oral cancer patients	Possible involvement in tumor microenvironment; exact role requires further investigation	(Pushalkar et al., 2012)
*Streptococcus salivarius*	Laboratory-based study	Presence in the oral cavity	Exhibits anti-inflammatory properties; potential therapeutic role in alleviating oral mucositis in cancer patients	(Kaci et al., 2014; Baek and Lee, 2023)
*Streptococcus intermedius*	Clinical	Isolated from cervical lymph nodes in oral cancer patients	Potential involvement in metastasis to cervical lymph nodes	(Sakamoto et al., 1999b, 1999a)
*Streptococcus constellatus*	Clinical	Increased abundance with cancer progression	Associated with advanced stages of OSCC; potential role in tumor progression	(Yang et al., 2018)
*Streptococcus oralis*	Clinical	Detected across all samples in OSCC patients	Part of the core bacteriome in OSCC patients; role in carcinogenesis not fully understood	(Sakamoto et al., 1999a; Chattopadhyay et al., 2019; La Rosa et al., 2020)
*Streptococcus sanguinis*	Clinical	Associated with a reduced risk of laryngeal cancer	Presence correlated with oral health; the mechanisms of the protective role require further study	(Hayes et al., 2018; Chattopadhyay et al., 2019)

Furthermore, we review key *Streptococcus* spp. and their interactions in oral cancer conditions, which are pivotal to oral cancer prognosis and manifestations. These include oral *Streptococcus* spp. from the mutans group, mitis group, anginosus group, and some other species of relevance, as discussed in the following sections.

##### 2.1.2.1 Streptococcus mutans

*S. mutans*, popularly known as the major contributor to tooth decay and root caries, is a facultative anaerobic opportunist of the human oral cavity. It is a well-known bacterium known for its role in inducing oral dysbiosis and modulating the oral health of an individual (Lemos et al., 2019). Recently, the association between *S. mutans* and OSCC has become a topic of increasing discussion and research. Several studies have found an increased association of *S. mutans* in oral cancerous microenvironments and have aimed to identify the mechanism by which this bacterium promotes disease progression (Tsai et al., 2022; Zhou et al., 2024). Studies have claimed that *S. mutans* is highly colonized in cancerous lesions (Xiao and Li, 2025). This contributes to the progression of OSCC by acidogenic and extracellular polymeric substance formation, triggering immune and chronic inflammatory responses (Senthil Kumar et al., 2024b).

*S. mutans* has positive implications in promoting the progression of oral cancer cells. This was proven by *in vitro* and *in vivo* experiments conducted by Tsai et al. (2022). The researchers effectively showed an increase in cell proliferation of SCC4 and SCC25 oral cancer cell lines infected with *S. mutans* after incubating for 8 hours, using the scratch assay (**Supplementary Figure S3a**). *S. mutans* also supported tumor metastasis in tumor-bearing mice, with incidences of lung metastasis of oral tumors noted by histological studies (**Supplementary Figure S3b**) (Tsai et al., 2022). However, it is interesting that not all *S. mutans* have the capacity to promote cell proliferation and migration. Alanazi et al. (2018) deciphered that infection of *S. mutans* isolates from prosthesis patients and cancer patients with prostheses promoted more adhesive potential and cell proliferative effects compared to infection with *S. mutans* isolated from healthy individuals. To elucidate this phenomenon at the molecular level, the researchers conducted several metabolome level and molecular analysis, which revealed that *S. mutans* isolates from cancer patients had significantly higher glycan and lactic acid production compared to isolates from healthy individuals. Additionally, genomic analysis of these cancer-patient-derived *S. mutans* strains revealed a higher prevalence of key virulence genes. For instance, 100% of isolates from the cancer-patient group carried genes for glucosyltransferases (*gtfB, gtfC*) and glucan-binding proteins (*gbpA-D*), which are all essential for biofilm formation, compared to only 45% of isolates from healthy controls. Furthermore, this was validated *in vitro* by co-culturing *S. mutans* isolated from healthy individuals, denture-wearing individuals, and cancer patients with buccal epithelial cells, which highlighted that *S*. *mutans* strains isolated from cancer patients had a significantly higher adherence capacity to epithelial cells than those isolated from healthy individuals. These findings provide a clear functional and genetic explanation for the strain-specific adaptations of *S. mutans* that enhance its pathogenic influence in the context of oral cancer, particularly among prosthesis-bearing and OSCC-affected individuals (Alanazi et al., 2018).

##### 2.1.2.2 Streptococcus mitis

*S. mitis* is one of the most isolated Mitis Group Streptococci (MGS), consisting of early oral cavity colonizers. These are commensal and non-pathogenic facultative anaerobes, known for their role in maintaining oral homeostasis by protecting the oral health of an individual (Abranches et al., 2018; Okahashi et al., 2022). *S. mitis* has been known to regulate inflammatory responses in the host only to the extent required for normal wound healing processes or immune responses against infection, not by inducing strong inflammatory responses leading to tissue damage or chronic effects (Engen et al., 2017, 2018).

In connection with oral cancer, it is deciphered that the abundance of *S. mitis* relatively decreases as oral cancer progresses, as shown through a metagenomic approach (Yang et al., 2018). However, it is worth noting that *S. mitis* exhibits anti-oncogenic effects. Transcriptomic research conducted by Baraniya et al. (2022) hypothesized that oral *S. mitis* has potential anti-inflammatory, anti-angiogenic, and antiproliferative effects *in situ*, essential for inhibiting carcinogenesis and metastasis. They demonstrated pro-apoptotic effects on oral cancer cells, resulting in tumor suppression. Additionally, this bacterium could inhibit the inflammatory and carcinogenic outcomes caused by other pathogenic bacteria associated with OSCC (**Supplementary Figure S4a**) (Baraniya et al., 2022). This followed an earlier study by Baraniya et al. (2020), where it was observed that hydrogen peroxide produced by *S. mitis* is responsible for *in vitro* cytotoxic action against oral cancer cells CAL27, SCC-25, and SCC-4 (**Supplementary Figure S4b**). The hydrogen peroxide pushes cells towards an apoptotic fate, thereby avoiding their proliferation and tumorigenesis (Baraniya et al., 2020). Another recent study by Inui et al. (2025) observed that *S. mitis* induces G2/M arrest and upregulates dual specificity phosphatase 1 (DUSP1), to demonstrate anti-proliferative effects in HSC-3 cells *in vitro* (Inui et al., 2025). The *Streptococcus*-DUSP1-HNSCC axis is one of the least explored host-microbial molecular pathways to date, to the best of our knowledge. However, the tumor-suppressive role of DUSP1, which counteracts key onco-supportive MAPK signals such as p38 and JNK, leading to G2/M arrest and apoptosis, has been well characterized and proven in various *in vivo* models. For instance, *in vivo* mouse models of head and neck cancer, as studied by Zhang et al. (2014), have demonstrated that DUSP1 deficiency in mice leads to enhanced disease progression, characterized by increased inflammation and elevated levels of pro-tumorigenic cytokine IL-1β (Zhang et al., 2014). Therefore, the finding that *S. mitis* can upregulate DUSP1 *in vitro* points to a significant potential mechanism for its anti-oncogenic effects; however, direct validation of this specific *S. mitis*-DUSP1 interaction in an *in vivo* oral cancer model remains a critical research gap. Although this bacterium exerts an anti-oncogenic effect, the exact mechanism by which its abundance decreases as OSCC progresses still remains a mystery.

##### 2.1.2.3 *Streptococcus anginosus* and *Streptococcus constellatus*

*S. anginosus* from the Streptococcus Anginosus Group (SAG) is a typical opportunistic inhabitant of the human oral, upper respiratory tract, gastrointestinal, and vaginal flora. They were considered a part of oral flora without any specific clinical manifestations for years. However, recently, they have been in the limelight because of their pathogenic influence, leading to life-threatening conditions (Pilarczyk-Zurek et al., 2022). Lately, studies have indicated their association, both progressive and regulatory, with oral cancer cases as well (Sasaki et al., 2005; Chocolatewala et al., 2010).

A recent study by Senthil Kumar et al. (2024a) elucidated the role of *S. anginosus* in exhibiting pro-inflammatory responses *in vitro* by the activation of NF-κB when co-cultured with macrophages, with elevated levels of IL-1β, IL-6, and TNF-α. The same study also suggested that infecting *S. anginosus* with macrophages induces inflammatory mediators such as inducible nitric oxide synthase (iNOS) and cyclooxygenase 2 (Cox2) in macrophages (Senthil Kumar et al., 2024a). The fact that these cytokines are known to have a positive correlation with cancer progression highlights the role of *S. anginosus* in the progression of oral cancer (Gelfo et al., 2020; Wu et al., 2025). However, in another research conducted by Xu et al. (2021), the researchers found that *S. anginosus* reduces the proliferation, migration, and invasion of tongue cancer cell line SCC15 and promotes cell apoptosis *in vitro*, uncovering the anti-oncogenic effects of *S. anginosus* (Xu et al., 2021). The paradoxical roles of *S. anginosus* are linked to both pro-inflammatory responses associated with cancer progression and anti-proliferative effects on oral cancer cells *in vitro*, highlighting the mechanistic complexity of host-streptococcal interactions in the context of oral cancer. Several factors might contribute to these differing observations, including (i) strain-level genetic diversities existing within *S. anginosus*, with the virulence and metabolic capabilities within the strains used in these different studies possibly leading to such distinct observations; (ii) *S. anginosus* might interact differently with immune cells versus epithelial cancer cells. The pro-inflammatory effects observed in the study by Senthil Kumar et al. (2024a) were reflected in macrophage co-culture assays, which indicate immune modulation. In contrast, the anti-proliferative effects reported by Xu et al. (2021) were demonstrated through direct infection of *S. anginosus* with SCC15, a tongue cancer cell line; (iii) The microbial milieu may vary in *in vitro* interaction studies and *in vivo* conditions. However, in the case of studies reporting paradoxical phenomena of *S. anginosus* in oral cancer, research by Senthil Kumar et al. (2024a) and Xu et al. (2021) both utilized the same variant of *S. anginosus* (ATCC 33397). Hence, we speculate that our hypothesis, which posits that the differential interaction of the bacteria with host immune cells and epithelial cells, holds true in this case. Still, there is a need for extensive studies on this bacterium to decipher the actual association and improve prognosis and patient outcomes in OSCC.

*S. constellatus* is another member of the SAG group, which is an inhabitant of the human oral cavity. Like *S. anginosus*, the relative abundance of *S. constellatus* is also positively correlated in OSCC cases and HNC cases (Kwak et al., 2024). The relative abundance of *S. constellatus* increased significantly in precancer and cancer conditions of the oral cavity (Yang et al., 2018). Although there are studies suggesting the role of *S. constellatus* in portraying inflammatory and septic signatures in affected cells, there is a need for experimental validation to decipher the mechanistic role of this bacterium in oral cancer (Abe et al., 2014).

##### 2.1.2.4 *Streptococcus sanguinis* and *Streptococcus gordonii*

*S. sanguinis* and *S. gordonii* are opportunistic oral pathogens that belong to the sanguinis group of *Streptococcus*. This group of streptococci are pioneer in colonizing the oral cavity of humans and are usually known for their infectious role in caries-associated conditions; however, their presence in and across the head and neck cancer etiologies is also notable (Kwak et al., 2024). While *S. sanguinis* abundance is found to decrease across the transition from a healthy state to oral cancer (Chang et al., 2019), studies suggest its pro-oncogenic effect in nasopharyngeal cancer by activating oncogenic Epstein-Barr Virus (EBV), through the production of hydrogen peroxide (H_2_O_2_). It was validated with increasing serum EBV VCA-IgA levels as the abundance of *S. sanguinis* increased (**Supplementary Figure S5**) (Liao et al., 2023). This contrasting role of *S. sanguinis*, decreased abundance across transition, and pro-oncogenic role linked to EBV activation, reflects distinct opportunistic interactions of the bacterium with the host tissue microenvironment. Further, specific interactions with viral co-factors, and other virulence factors leading to oncogenic outcomes should be explored in future.

On the other hand, *S. gordonii* has shown anti-oncogenic effects by downregulating the *Porphyromonas gingivalis*-mediated induction of ZEB2, which is required for the proinflammatory, mesenchymal-like transcriptional program in host tissue (Ohshima et al., 2019; Fitzsimonds et al., 2020). *S. gordonii* also produces hydrogen peroxide, which is cytotoxic in nature and promotes cellular apoptosis in oral cancer cells (Zheng et al., 2011; Lin et al., 2019). However, the mechanism and its ill effects leading to EBV activation, as compared with to that of *S. sanguinis*, are unknown for *S. gordonii*. Additionally, *S. gordonii* is also known to co-aggregate with pro-oncogenic pathogens, such as *Fusobacterium nucleatum* (Yang et al., 2022). Hence, the role of *S. gordonii* in the oral cancer microenvironment completely depends on the context of its surrounding microbial community.

## 3 Mechanisms of streptococcal involvement in oral cancer

The involvement of *Streptococcus* spp. in oral cancer is multifaceted due to its inherent properties, such as its potential to form biofilms, ability to co-aggregate with other microbes, immunomodulatory potential, virulence factors, and secretion of some metabolites involved in both pro- and anti-carcinogenic mechanisms. Understanding these mechanisms of interaction and survival within the oral cavity of the host is essential to decipher the interplay of oral streptococci associated with oral cancer.

### 3.1 Biofilm formation and co-adhesion strategies associated with oral streptococci

**Figure 3:**
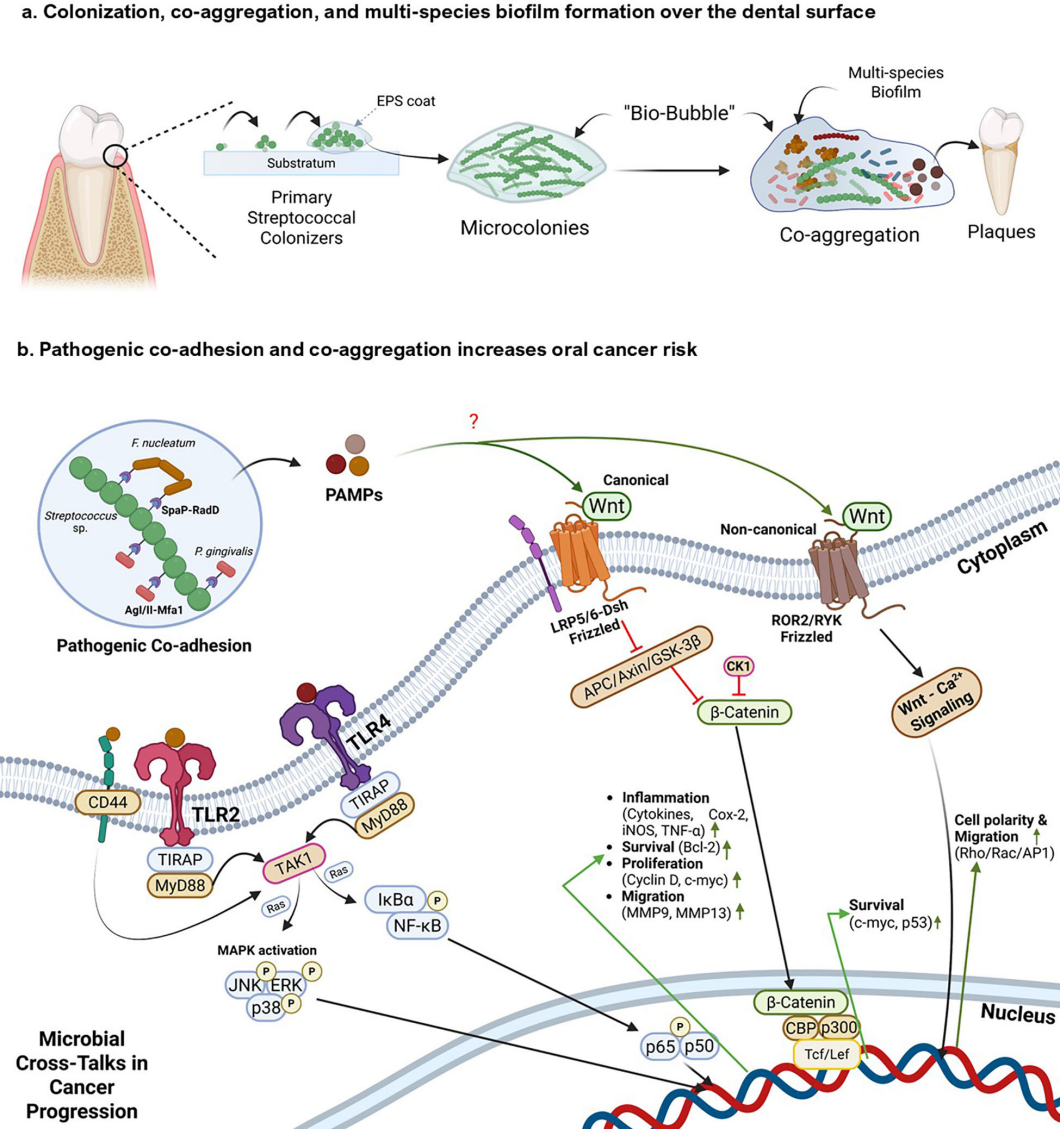
Mechanistic insights of streptococcal co-aggregation in oral cancer progression **(a)** depicts colonization and multispecies biofilm formation by oral streptococcal species. Primary streptococcal colonizers bind to the dental substratum and create an Extracellular Polymeric Substance (EPS) matrix, establishing microcolonies and a protective ‘bio-bubble’. These formations facilitate co-aggregation with secondary colonizers, creating a multi-species biofilm that develops into dental plaque. **(b)** This panel illustrates how interactions between pathogenic bacteria within the biofilm trigger pro-oncogenic host signaling pathways: i) Initial interaction occurs between *Streptococcus* sp. and pathogenic bacteria such as *F. nucleatum* and *P. gingivalis* through receptor-mediated co-adhesion and co-aggregation (e.g., SpaP-RadD, AgI/II-Mfa1). ii) These interactions, along with general microbial Pathogen-Associated Molecular Patterns (PAMPs), activate host pattern recognition receptors such as Toll-like receptors (TLR2/4) and cell surface markers such as CD44. iii) TLR activation initiates downstream signaling cascades, including the MAPK and the NF-κB pathway, which drive the expression of genes involved in inflammation, cell survival, proliferation, and migration. iv) Additionally, bacterial PAMPs can als modulate host Wnt signaling. The canonical Wnt pathway leads to the inhibition of the β-catenin destruction complex. This results in β-catenin accumulation, nuclear translocation, and activation of Tcf/Lef transcription factors, promoting expression of oncogenic targets such as *Cyclin D1* and *c-Myc*. Non-canonical Wnt signaling can also be activated, influencing cell polarity and migration. Created using BioRender.

Oral *Streptococcus* spp. play an important role in the early colonization of the oral cavity. Commensal species such as *S. gordonii, S. infantis, S. mitis*, and *S. oralis* are involved in early colonization of the oral cavity by forming biofilms on the surfaces of the tooth pellicle, a structure formed by salivary glycoproteins on the surface of the dental enamel (Smith et al., 1993; Socransky et al., 1998; Nobbs et al., 2009; Pimenta et al., 2019). Oral streptococci make use of glycoprotein binding surface receptors such as Ag I/II family receptors and SsaB receptors to bind with salivary glycoproteins such as gp340, proline-rich salivary proteins, and α-amylase from the saliva, which helps them to colonize the oral environment (Nobbs et al., 2009). There are several other uncharacterized receptors that also mediate streptococcal adherence to dental surfaces. After adherence, they start to aggregate in four progressive stages – (i) formation of the primary colonies, (ii) formation of microcolonies, (iii) co-aggregation and co-adhesion, and (iv) multi-species biofilm formation stage (**Figure 3a**) (Rickard et al., 2003). Initially, oral streptococci form their primary colony on the tooth surface, which later starts to produce extracellular polysaccharides (EPS). This EPS mediates the formation of microcolonies by forming a thin protective film around the colonies of these bacteria, creating a ‘bio-bubble’ inside which the bacteria rapidly grow and divide, thereby increasing their population. This bio-bubble helps the primary colonizers to resist stressful conditions, such as the effect of organic acids in the saliva, salivary lytic enzymes like proteases and amylases, and even stressful conditions including colony erosion due to eating patterns that might destroy the primary colonies (Abranches et al., 2018). Later, as the population of initial streptococcal colonizers increases, other commensals bind to the surface receptors of these bacteria and start to form a multispecies biofilm (Rickard et al., 2003). In a healthy state, the multispecies biofilm is characterized only by oral commensals and a few opportunists. It is also evident that these commensal biofilms hinder the pathogenic growth in the oral cavity. However, poor oral hygiene practices, poor diet habits, and lifestyle changes lead to a pathogenic rush inside the oral cavity, which enables the pathogen to evade and colonize within these multispecies biofilm communities (Kolenbrander et al., 2010). These pathogenic multispecies biofilms cause oral dysbiosis, starting the tale of endless infectious triggers. It begins with the infection of the gums, causing gum abscesses and gingivitis, gradually leading to localized bacteremia and periodontitis (Rickard et al., 2003; Bowen et al., 2018).

Primary streptococcal colonizers possess distinct surface receptors mediating multispecies biofilm formation (Nobbs et al., 2009; Abranches et al., 2018). **Supplementary Table S1** provides a detailed summary of various surface receptors of oral streptococci involved in biofilm formation and co-aggregation, implicating their responses in the host oral cavity. Streptococcal aggregation with some pathogenic bacteria also increases the risk of oral cancer and its progression (**Figure 3b****).**Yang et al. (2022) investigated the ability of *F. nucleatum*, a bacterial pathogen known to have a role in oral carcinogenesis and progression, to co-aggregate with oral commensal *S. gordonii*, and explored its virulence in co-aggregated and co-infected groups by co-culturing with human gingival epithelial cells (hGECs). The gene expression levels of TLR2 and TLR4 in hGECs increase during co-aggregation, compared to those in infection with monocultures. It was also observed that co-aggregation inhibited apoptosis of hGECs and promoted secretion of pro-inflammatory cytokines such as TNF-α and IL-6 in hGECs; interestingly, the secretion of the anti-inflammatory cytokine TGF-β1 was inhibited. Coaggregation was also seen to phosphorylate p65, p38, and JNK proteins and activate NF-κB and MAPK signaling pathways, hallmarks of tumor migration (Yang et al., 2022). The biofilm-mediated cytokine expression leads to a pro-inflammatory and pro-tumorigenic microenvironment. A study by Guo et al. (2017) reported that *F. nucleatum* subsp. *polymorphum* can adhere to *S. mutans* through *F. nucleatum* RadD – *S. mutans* SpaP outer surface receptor interactions (Guo et al., 2017). Lima et al. (2017) also reported the co-adherence of *F. nucleatum* to *S. gordonii* through co-aggregation mediated by protein A (CmpA) (Lima et al., 2017). Moreover, He et al. (2012) hypothesized that it is the adherence capacity of *F. nucleatum* to oral streptococcal species that mediates its integration into the oral microbial community (He et al., 2012).

Though *Streptococcus* spp. are known to be early colonizers, some species cannot invade oral epithelial cells. Such species depend on other bacteria (e.g., *F. nucleatum*) to internalize themselves into host cells (Edwards et al., 2006). Edwards et al. in 2006 and 2007 reported that *F. nucleatum* internalizes late colonizing *S. cristatus* to hGECs through the RadD binding mechanism and a high molecular weight arginine binding protein (Edwards et al., 2006, 2007). Other bacterial players involved in OSCC, such as *P. gingivalis*, also use a co-aggregation mechanism to be integrated into the oral microbial community. Daep et al. (2008) reported that streptococcal antigen I/II interacts with the minor fimbrial antigen (Mfa1) of *P. gingivalis* for co-aggregating within the oral cavity. They also reported the conserved peptide sequence in the BAR protein derived from streptococcal antigen I/II, which contains the sequence KKVQDLLKK, as responsible for mediating *P. gingivalis* adherence to *S. oralis*. The study also identified another conserved sequence, VQDLL, that forms an amphipathic α-helix structurally similar to the NR box motif (LXXLL) found in eukaryotic nuclear receptors, which mediates protein-protein interactions. These interactions interfere with the ability of *P. gingivalis* to adhere to oral streptococci, which is key for its biofilm formation in the oral microenvironment (Daep et al., 2008).

Apart from direct inflammatory responses signaling through co-aggregation strategies, pathogenic biofilms reshape the tumor microenvironment by producing several biofilm-associated metabolites. A prime consequence of biofilm-associated metabolites is the localized reduction in pH, mediated by the biofilms in the oral cavity. Cariogenic bacteria such as *S. mutans* and *S. sobrinus* form biofilms, which reduce the pH locally in the oral cavity by producing acidogenic metabolites by fermenting carbohydrates (Schultze et al., 2021). The acid microenvironment is positively correlated with the progression of cancer (Boedtkjer and Pedersen, 2020). Cancer cells also produce organic acids such as lactate, which inhibit the anti-tumor response mediated by innate and adaptive immune cells. This favors tumor progression and reduces the response to immunotherapy (Cappellesso et al., 2024). The acidic microenvironment also supports the growth of opportunists such as *S. mutans*, having the ability to co-aggregate and form biofilms (Matsui and Cvitkovitch, 2010). Thus, an intricate interplay between onco-microbial symbiosis leads to synergistic progression and growth of cancer cells and pathogens *in situ*, increasing the risk of malignancy and metastasis by several folds. However, it is also worth noting that these pathogenic co-aggregations and inflammatory responses compromise the host epithelial barrier. Persistent acid production by pathobiont *Streptococcus* sp. not only disrupts and demineralizes tooth enamel, but it can also damage epithelial cells and disrupt tight junctions (Şenel, 2021). This, along with the potential secretion of bacterial enzymes such as hyaluronidases and proteases, leads to the breakdown of the epithelial barrier, which in turn facilitates microbial translocation, sustained immune activation, and deeper tissue invasion (Bloch et al., 2024). This creates a feed-forward loop that promotes chronic inflammation and oncogenic transformation. On the contrary, some commensal species, such as *S. salivarius*, have developed urease systems that can modulate the pH of the oral cavity by creating an alkaline microenvironment. This also inhibits the co-aggregation of pathogenic bacterial populations, such as *S. mutans* and *A. actinomycetemcomitans*, thereby lowering the risk of multispecies pathogenesis. Producing an alkaline microenvironment can also be correlated with an increased immune response, which is associated with elevated anti-biofilm and anti-tumor properties (Begić et al., 2023). While these commensal biofilms might create homeostasis in the oral microenvironment, the focus specifically lies on pathogenic biofilms, which might trigger inflammatory responses in the oral microenvironment conducive to carcinogenesis and progression.

### 3.2 Inflammatory responses associated with oral streptococci and their manifestations in oral cancer

Some pathogenic and opportunistic oral streptococci significantly impact the immune responses of the host in several ways, leading to carcinogenesis, tumor growth, malignancy, or a weakened immune system unable to combat cancer. Chronic inflammation is often referred to as the ‘seventh hallmark of cancer’ because of its ability to drive tumorigenesis, cancer cell proliferation, and metastasis (Balkwill and Mantovani, 2001; Milane, 2022). Several species of streptococcal pathobionts, particularly *S. mutans* and *S. anginosus*, are directly involved in inducing inflammatory responses in oral cancer cells. Tsai et al. (2022) found that infection by *S. mutans* causes an elevation in the levels of IL-6 in 4NOQ-induced mice. It is to be noted that the IL-6/STAT3 pathway plays a critical role in cancer progression and epithelial-mesenchymal transition (EMT) that leads to increased migration of the cancer cells. This pathway has also been implicated in various microbe-induced carcinogenesis and tumor progression. Infection of *S. mutans* with the oral cancer SCC4 cell lines led to elevated levels of β-catenin and matrix metalloproteinase-9 levels, which suggests the migratory potential of the cells by detaching from ECM, implicated due to reduced E-cadherin expression, a cell-adhesion molecule that plays a critical role in fixture of cells to the ECM (**Figure 3b**). *S. mutans* infection to SCC4 cells also increased the levels of CD44 and acetaldehyde dehydrogenase 1 (ALDH1), both of which are cancer stem cell markers (Tsai et al., 2022). CD44 is a transmembrane glycoprotein that helps tumor progression and metastasis by enabling cell adhesion, movement, and interaction with the surrounding tissue through binding to hyaluronic acid (Zhu et al., 2025). It activates Ras-MAPK, PI3K-Akt, and Wnt/β-catenin signaling pathways, which boost cell growth, survival, and EMT to support cancer stem cells (Chen et al., 2018). ALDH1 is another known marker for cancer stem cells that activates several pathways, including the USP28/MYC signaling pathway, the ALDH1A1/HIF-1α/VEGF axis, and the Wnt/β-catenin signaling pathway (de Freitas Filho et al., 2021; Wei et al., 2022). These pathways lead to the progression of cancerous cells by providing them with a perfect molecular niche to grow, divide, and multiply rapidly. ALDH1 also helps regulate the internal pH of cells, which aids in their survival and resistance to treatment (Wei et al., 2022). Yu et al. (2024) reported that *S. mutans* infection leads to thrombosis formation, further supporting tumor metastasis. This study suggested that *S. mutans* stimulates endothelial cells to exhibit various hallmark inflammatory pathways in cancer progression. They reported the activation of interferons, CD40 signaling, platelet aggregation, and coagulation, which promoted a pro-inflammatory, pro-thrombotic environment potentially relevant to cancer and vascular disease progression (Yu et al., 2024).

*S. anginosus* is another oral *Streptococcus* with inflammatory cross-talks in oral cancer progression. Senthil Kumar and group performed co-culture assays of *S. anginosus* and *S. mitis* with RAW 264.7 macrophages. They found that after coculturing, the macrophages exhibited elevated levels of pro-inflammatory cytokines, such as TNF-α, IL-6, and IL-1β, when infected with *S. anginosus*; however, this phenomenon was not observed when macrophages were infected with *S. mitis*. In addition, the levels of anti-inflammatory cytokines such as IL-10 were downregulated (Senthil Kumar et al., 2024a). Whereas IL-6 and TNF-α are involved in cancer cell invasiveness and metastasis, IL-1β has pleiotropic effects on immune cells and supports the survival and progression of cancer cells by promoting angiogenesis, proliferation, migration, and metastasis (Balkwill, 2009; Rébé and Ghiringhelli, 2020; Rašková et al., 2022). This mechanistic interplay between pathogenic streptococci plays a significant role in modulating the cancer microenvironment, which promotes cancer progression.

While the pro-inflammatory role of *S. anginosus* and related species is evident in oral carcinogenesis and progression, some studies also reveal its anti-proliferative milieu in cancer cells. Xu et al. (2021) reported that *S. anginosus* promoted cellular apoptosis in SCC15 cells, reducing the potential of SCC15 cells to proliferate, migrate, and invade. The researchers also confirmed the involvement of the bacterium in inducing autophagy to cause cell death in SCC15 cells, by confocal microscopy involving monodansylcadaverine staining (Xu et al., 2021). However, the exact molecular mechanism underlying this phenomenon has not been reported.

### 3.3 Streptococcal-mediated metabolic reprogramming in oral cancer

**Figure 4:**
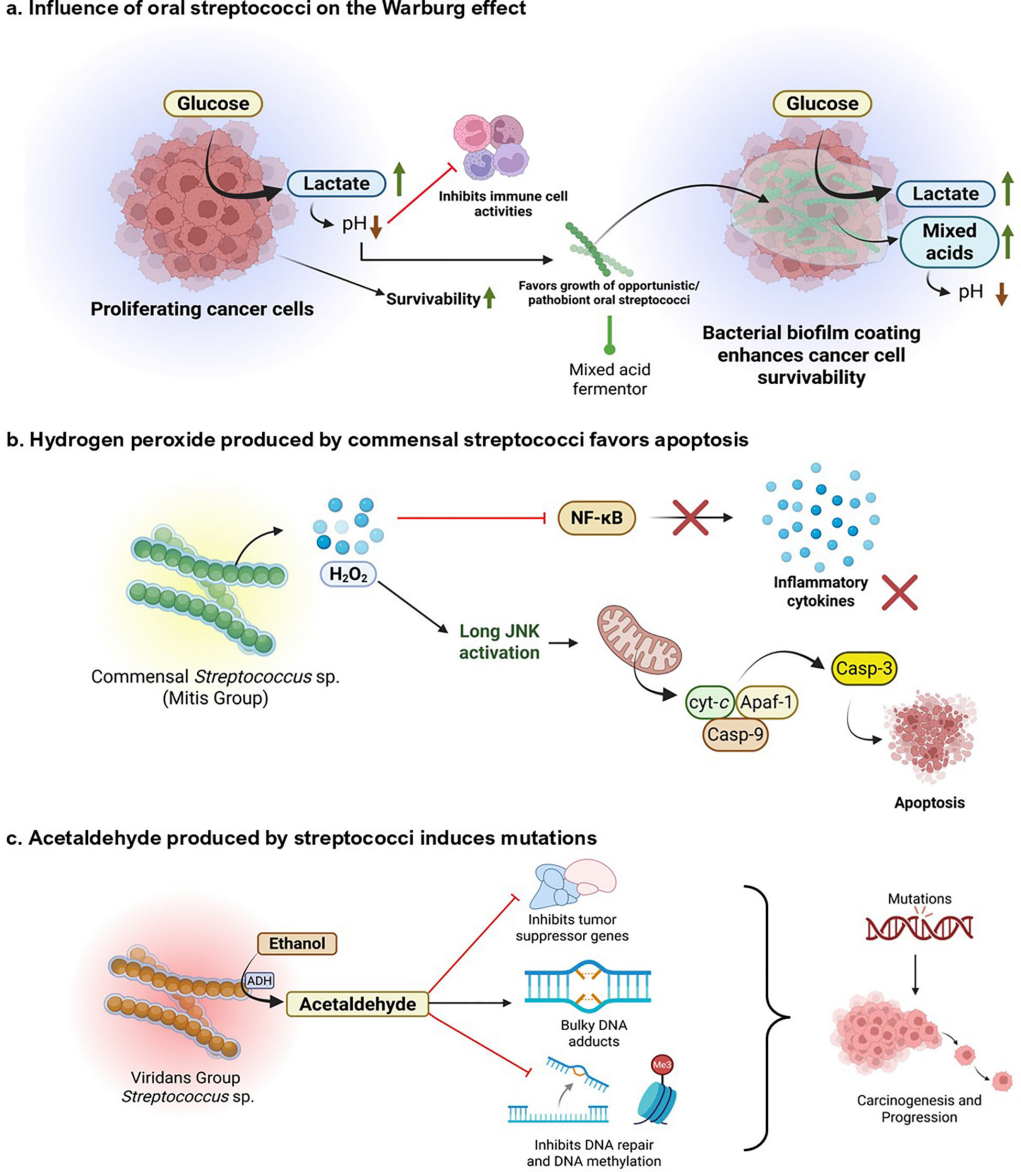
Metabolic and genotoxic roles of oral *Streptococcus* species associated with oral cancer. **(a)** The impact of Warburg effect. Proliferating cancer cells display increased glucose consumption and lactate production, reducing pH and compromising immune cell function. This acidic, immunosuppressed microenvironment increases cancer cell survival and favors colonization with opportunistic/pathogenic Streptococcus species. Mixed acid fermentation by these pathogens further acidifies the environment, enforcing tumor growth and microbial persistence through biofilm development. **(b)** Commensal *Streptococcus* (Mitis Group) generate H_2_O_2_, which stimulates the JNK pathway and triggers mitochondrial apoptosis via cytochrome c release and caspase-3 activation. This suppresses NF-κB signaling and downstream inflammatory cytokines, implying a protective, pro-apoptotic function against tumorigenesis. **(c)** Ethanol metabolism by the viridans group Streptococcus results in the accumulation of acetaldehyde, which disrupts the expression of tumor suppressor genes, induces bulky DNA adducts, and inhibits DNA repair and methylation. These genotoxic actions lead to the accumulation of mutations, which stimulate oral carcinogenesis and tumor growth. Created using BioRender.

While *Streptococcus* spp. is pivotal in mediating pro-inflammatory responses associated with oral cancer cell lines, their metabolites are equally notable in regulating cancer progression (**Figure 4****).***Streptococcus* spp. is involved in various metabolic events that can support or impede cancer progression. The different metabolic events by *Streptococcus* spp. that play an essential role in oral cancer manifestations are discussed as follows.

#### 3.3.1 Warburg effect and pH regulation

Warburg effect involves aerobic glycolytic shifts adopted by cancer cells to convert glucose into lactate, even in the presence of sufficient oxygen. Compared to the longer oxidative phosphorylation mechanism, this helps the cancer cells generate sufficient energy immediately and yield metabolically important byproducts necessary for synthesizing nucleotides, fatty acids, and amino acids essential for cell growth and proliferation (Liberti and Locasale, 2016). Various oral bacteria, including *S. mutans* and related species, convert carbohydrates to lactate through mixed acid fermentation. This mimics the symbiotic Warburg effect, where bacteria get the surface to adhere and nutrients from the cancer cell, and the cell gets metabolic byproducts, energy, and lactate in return, creating a conducive setting for cancer progression (Duguid, 1985; Dashper and Reynolds, 1996; Krzyściak et al., 2014; de la Cruz-López et al., 2019).

Various *Streptococcus* spp. are involved in producing organic acids through carbohydrate metabolism, including lactate, acetate, and butyrate, which promote the growth of acid-tolerant bacteria (Liu et al., 2020; Wang et al., 2021; Xu et al., 2025). Acid production also protects the bacterial colonies within the biofilm matrix from host immune responses (Xu et al., 2025). However, cancer cells take advantage of this acidic environment to harness their growth and impede cytotoxic immune responses. This leads to growth, proliferation, migration, and metastasis of cancerous cells (Boedtkjer and Pedersen, 2020).

#### 3.3.2 Hydrogen peroxide production

Most of the commensal *Streptococcus* spp., including the MGS, are alpha-hemolytic in nature, meaning they can produce H_2_O_2_ (Tang et al., 2022). Hydrogen peroxide, known to induce several metabolic alterations in cancer cells, plays a dual role in cancer. While H_2_O_2_ can affect both cancerous and non-cancerous cells by inducing oxidative stress, its effects are often more pronounced in cancer cells due to their already elevated levels of reactive oxygen species (ROS) and altered redox balance. It is involved in pro-oncogenic activities by causing DNA alterations, promoting angiogenesis and metastasis, and activating hypoxia-inducible factor 1 (HIF-1). It is also involved in the induction of apoptosis in cancer cells, halting their progression (López-Lázaro, 2007). Some studies suggest the cytotoxic property of H_2_O_2_ produced by oral streptococci on macrophages (Okahashi et al., 2013). However, a significant research gap exists regarding the mechanistic role of bacterial-originated H_2_O_2_ and its effects on oral cancer environments. Future studies could focus on gathering mechanistic data on host signaling pathways modulated by streptococcal-derived H_2_O_2_, clarifying its concentration-dependent hormesis, to determine whether the niche could allow cancer cells to proliferate or undergo apoptosis. Additionally, the influence of bacterial-derived H_2_O_2_ on tumor-associated immune cells should be elucidated. Moreover, all these gaps should be correlated clinically to obtain a comprehensive mechanistic understanding of how streptococcal-derived H_2_O_2_ modulates oral cancer outcomes in patients.

#### 3.3.3 Acetaldehyde production

Some oral streptococci, particularly from the viridans group, are capable of converting ethanol into acetaldehyde during their metabolism. They have NAD-linked alcohol dehydrogenase (ADH) systems that convert alcohols into aldehydes (Nosova et al., 1997; Kurkivuori et al., 2007). A prominent study by Väkeväinen et al. (2021) has shown that acetaldehyde production in the oral cavity following alcohol consumption is primarily of microbial origin, as inhibiting human ADH does not significantly alter salivary acetaldehyde levels (Väkeväinen et al., 2001). Several common oral *Streptococcus* sp., including commensals such as *S. salivarius, S. intermedius, S. gordonii*, and *S. mitis*, possess significant ADH activity and produce high amounts of acetaldehyde when incubated with ethanol (Kurkivuori et al., 2007). Acetaldehyde is a known carcinogen, mutagen, and toxic agent. Acetaldehyde forms DNA adducts, such as N²-ethyl-2-deoxyguanosine (N²-Et-dG) and 1, N²-propano-dG (PdG), which cause bulky adduct defects and mutations in DNA. It is also involved in the enzymatic inhibition of O^6^ methyl-guanyl transferase, which is quintessential for repairing bulky adduct-induced DNA damage. Through DNA damage, acetaldehyde induces cell cycle dysregulation and activates oncogenic signaling pathways such as MAPK and NF-κB. The resultant genomic instability leads to carcinogenesis and progression (Seitz and Stickel, 2010). This observation can be attributed to lifestyle and habits correlated with an increased risk of cancer, such as an increased risk of dysbiosis in the oral cavity of individuals who frequently consume alcohol. Such individuals are more susceptible to oral cancer due to the aldehyde-producing property of oral bacteria (Fan et al., 2018). Apart from the bacteria-derived acetaldehyde, smoking, tobacco chewing, and vaping also introduce acetaldehyde into the host system, which adds to the inherent acetaldehyde production by oral bacteria, increasing the risk of oral cancer initiation and progression (Abiko et al., 2023; Shehata et al., 2023; Tackett et al., 2024).

It is interesting to note the paradoxical role of *Streptococcus* sp. in promoting oral cancer by producing acetaldehyde. For instance, *S. mitis* is a significant producer of acetaldehyde; however, it is typically associated with a healthy oral microbiome and tends to decrease as OSCC progresses, as discussed earlier. However, to better understand the specific contribution of acetaldehyde produced by bacterial ADH compared to that from external sources, such as alcohol consumption or smoking, targeted epidemiological studies are necessary. These studies should aim to connect oral microbial ADH profiles with the risk of OSCC to effectively understand the relationship between them.

#### 3.3.4 Metabolite regulations

**Figure 5:**
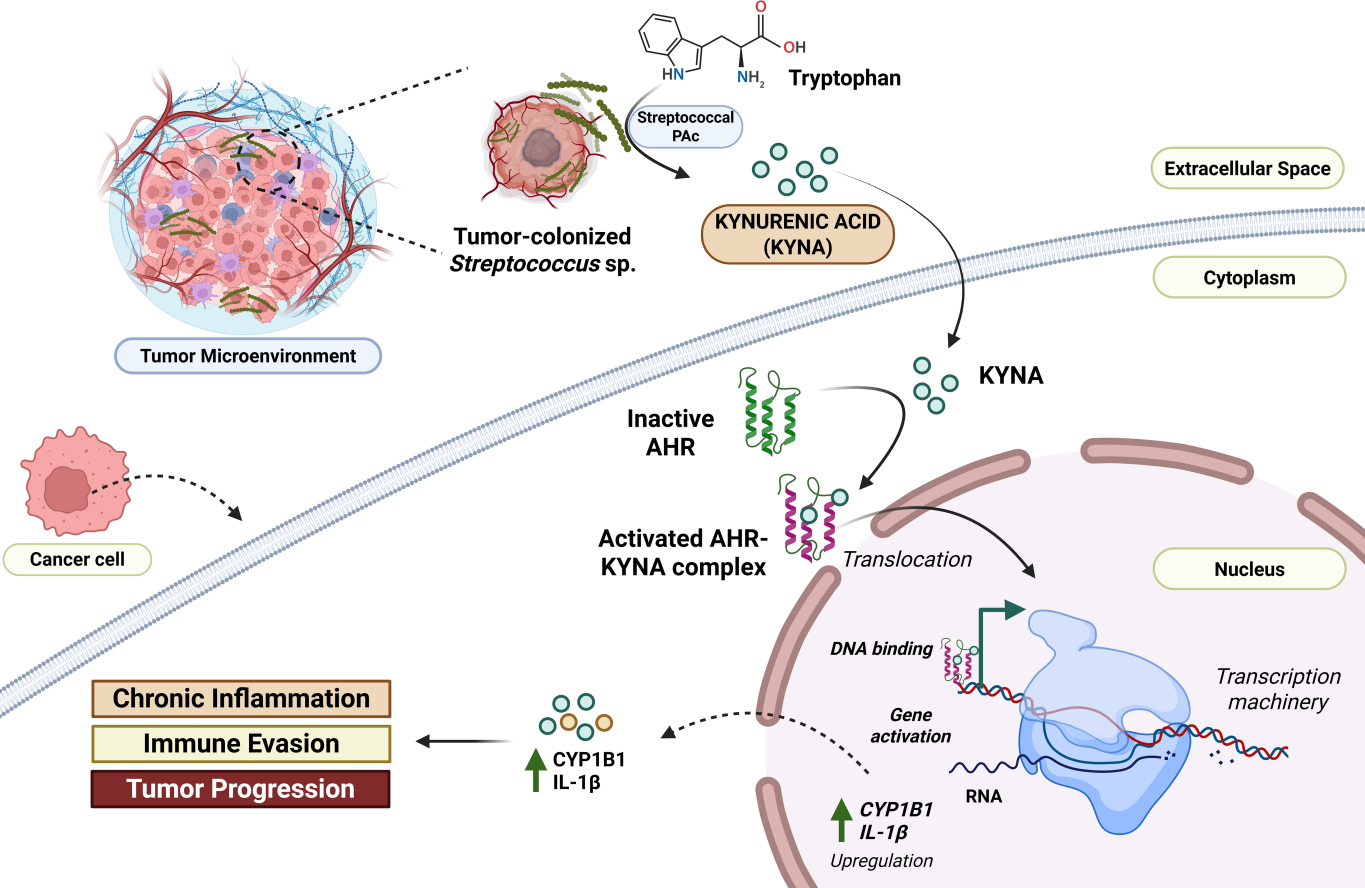
Conceptual understanding of *Streptococcus-*mediated metabolic reprogramming in the context of oral cancer. *S. mutans* and related *Streptococcus* sp. possess surface receptors such as Protein Antigen c (PAc). It converts the tryptophan into kynurenic acid (KYNA) in the tumor microenvironment. This KYNA enters the adjacent host cancer cells and binds to aryl hydrocarbon receptors (AHR). The activated KYNA-AHR complex translocates into the nucleus, promoting the upregulation of key pro-oncogenic genes, such as CYP1B1, and the pro-inflammatory cytokine IL-1β. The resulting increase in CYP1B1 and IL-1β contributes to chronic inflammation, immune evasion, and ultimately, tumor progression within the oral cancer microenvironment (Xiao and Li, 2025). Created using BioRender.

The role of *Streptococcus mutans* in inflammatory responses is well known. However, it also plays a significant role in the metabolic reprogramming of the oral tumor environment, enabling the production of onco-supportive metabolites. Zhou et al. (2024) reported that oral *S. mutans* isolated from oral cancer is associated with higher levels of kynurenic acid (KYNA) in the host salivary metabolome. They reported that *S. mutans* mediates the production of KYNA by utilizing its Protein Antigen c (PAc). They co-cultured SCC1 cells with PAc and KYNA to validate this and checked for their proliferative effects. It was found that KYNA, a tryptophan metabolite, is involved in supporting the tumor environment by expression of Solute Carrier family 7 member 5 (SLC7A5) and Solute Carrier family 7 member 8 (SLC7A8) transporters, which elevates the metabolic intake in cancer cells. They also reported the activation of the aryl hydrocarbon receptor (AHR) pathway, evidenced by elevated CYP1B1 expression in tumor tissues. KYNA was also linked to increased IL-1β expression, a pro-inflammatory cytokine driving inflammation (**Figure 5**) (Zhou et al., 2024). It is worth noting that KYNA originates from the kynurenic metabolism pathway (KP), which is linked with tumor progression and metastasis. The KP involves various enzymes such as indoleamine-2,3-dioxygenase 1 (IDO1), IDO2, and Trp-2,3-dioxygenase (TDO), which are crucial for tryptophan metabolism; however, they are linked to increased tumor immune resistance by depleting tryptophan or activating AHR pathway. The IDO1 is also linked with inducing pro-inflammatory responses. Together, these metabolic reprogramming can promote cancer progression by regulating NAD+ metabolism, enhancing angiogenesis and metastasis, and inhibiting tumor ferroptosis. These mechanisms are crucial to decipher bacterial pathogenesis in oral cancer pathophysiology (Lu et al., 2025).

## 4 Transition from normal to cancerous state – *Streptococcus* spp. as biomarkers in oral cancer

The transition from a normal to a malignant condition in the oral cavity is also associated with profound alterations in the oral microbiome. *Streptococcus* spp. have been identified as promising microbial biomarkers for the early detection of oral cancer. Clinical studies have shown that species such as *S. anginosus*, *S. mutans*, and *S. gordonii* are overrepresented in OSCC tissues relative to healthy or adjacent non-tumorous tissues (Torralba et al., 2021; Tsai et al., 2022; Zhou et al., 2024). Their involvement in inflammation, DNA damage, and the production of carcinogenic metabolites makes them promising candidates for developing diagnostic markers, even by adapting point-of-care strategies to detect their presence and rapidly decipher oral health. Conversely, species from the commensal mitis and sanguinis group of streptococci, which are known to decline in OSCC development, can be used as indicators of a healthy oral microbiota (Yang et al., 2018; Chang et al., 2019). Advanced molecular approaches, such as 16S rRNA sequencing, metagenomics, and FISH/qPCR-based methodologies, have enabled the detection of these changes, shedding light on the microbial dynamics of oral carcinogenesis. Accordingly, longitudinal follow-up of streptococcal species within oral swabs or biopsies may be more valuable for improving risk stratification and aiding early detection of oral cancer, even in its pre-malignant or benign stages.

## 5 Potential therapeutic strategies associated with oral streptococcal dynamics

### 5.1 Targeting co-aggregation and biofilm formation

Interfering with co-aggregation and biofilm formation between oral streptococci can be a strategic point of target to manage oral cancer and its prognosis. Surface adhesins such as CshA/B in *S. gordonii* or VisA in *S. gordonii* that facilitate bacterial attachment to host tissue and co-aggregation with other microorganisms such as *Veillonella parvula* and *F. nucleatum* could be used as clinical targets (Dorison et al., 2024). The use and administration of anti-adhesion peptides, small molecule inhibitors, or antibody-based means can prevent the maturation of biofilms, decrease microbial synergy, and impede carcinogenic partner colonization (Gao et al., 2024). For example, synthetic BAR peptides that inhibit CshA/B activity have promise in preventing co-aggregation (Daep et al., 2008). Inhibiting biofilm integrity not only reduces the microbial burden but also suppresses local inflammation and carcinogen production. Moreover, quorum-sensing inhibitors of streptococcal communication networks (e.g., ComDE system) may decrease virulence expression and biofilm adhesion (Zu et al., 2019). These approaches, when combined with conventional treatments, may enhance oral cancer treatment by targeting the microbial niche that facilitates tumor growth.

### 5.2 Can probiotic intervention by beneficial streptococci be effective?

Probiotic treatment with commensal *Streptococcus* spp., such as *S*. *salivarius* K12 and M18 strains, is emerging as a method to reestablish oral microbial homeostasis lost in OSCC. Vesty et al. (2020) and Park et al. (2023) conducted such studies to investigate whether probiotic *S. salivarius* can colonize mucosal surfaces and exclude pathogenic equivalents through the production of bacteriocins, competition for nutrients, and immune system modulation (Vesty et al., 2020; Park et al., 2023). *S. salivarius* has shown anti-inflammatory activity and the capacity to decrease volatile sulfur compounds and carcinogenic virulent factors like arginine-specific gingipain A (RgpA) by *P. gingivalis* in the oral cavity (Park et al., 2023). Probiotics may also suppress biofilm formation and reduce epithelial barrier disruption, thereby limiting microbial translocation and tumor-supporting inflammation. Routine oral probiotic administration might act as an adjuvant to standard treatments by modulating the microbial profile to a more protective, anti-oncogenic state (Meroni et al., 2021; Lekshmi Priya et al., 2025). Such healthy strains incorporated in oral care products or supplements could provide a preventive approach, particularly in high-risk subjects with dysbiotic oral microbiota.

### 5.3 Targeting streptococcal-driven inflammation

Chronic inflammation has been established as a driving force behind oral cancer progression. Streptococcal species such as *S. mutans* and *S. anginosus* can induce intense inflammatory reactions through lipoteichoic acid (LTA), peptidoglycan fragments, and cytokine induction pathways (Tsai et al., 2022; Senthil Kumar et al., 2024a). Immunomodulatory therapy to target these microbial triggers is very important for managing oral cancer cases. Approaches that involve anti-inflammatory drugs to block IL-6, TNF-α, or CD40 signaling caused by streptococcal components can help manage oral cancer linked to dysbiosis. Recent studies have shown that when *S. mutans* stimulates endothelial cells, it activates the interferon and CD40 signaling pathways, which play roles in the tumor microenvironment (Yu et al., 2024). By neutralizing these inflammatory processes, angiogenesis and metastatic potentials can be inhibited; immune suppression can be combated, thus targeting bacterial-driven oncogenesis. Thus, combining treatments that focus on inflammation with efforts to eliminate microbes may provide a more comprehensive strategy for managing oral cancer.

## 6 Future directions and research gaps

Despite growing evidence associating *Streptococcus* spp. with oral cancer, various gaps in research impede an understanding of their mechanistic functions. Firstly, most current studies are cross-sectional, which constrains the understanding of causality between streptococcal changes and the initiation of oral carcinoma. Longitudinal studies on the follow-up of microbiome dynamics from premalignant lesions to carcinoma are necessary. Secondly, species-level resolution is usually insufficient; subspecies or strain-specific behavior (e.g., virulence, metabolite production) is under investigated due to limitations in existing sequencing methods. Thirdly, the interactions between *Streptococcus* and other microbial phyla, as well as the host epithelial and immune cells, need to be elucidated at the molecular level, including the integration of multi-omics data. These can be achieved through techniques such as spatial transcriptomics and single-cell omics studies, which can reveal host-streptococcal crosstalk. Along with these, strategies such as identifying key *Streptococcus* sp. as biomarkers for OSCC, derived from larger patient cohorts, and using them to target oral cancer therapeutics by employing emerging techniques such as phage-based targeting and species-specific immunotherapy will definitely aid in designing a streptococcal-based precision theragnostic approach for oral cancer. Such studies will clarify the commensal-pathogenic duality and may help identify novel theragnostic directions. Moreover, the roles of lifestyle, diet, and oral hygiene in influencing streptococcal behavior and their impact on cancer development are also poorly understood. Theragnostic approaches targeting streptococci are in the early stages, with clinical trials needed to detect, evaluate, and assess the safety and effectiveness of probiotics, phage therapy, and microbial-targeted adjunctive treatments. Lastly, integrating microbial biomarkers into diagnostic pathways for detecting oral cancer at an early stage has untapped potential. In addition to these, AI-based predictive machine learning models can be utilized for detecting microbial alterations associated with oral cancer risks and may be incorporated into future clinical and metagenomic studies. Furthermore, integrating lifestyle factors (such as alcohol consumption and smoking) into these predictive models could significantly enhance their accuracy for improved risk stratification. Filling these research gaps by integrating interdisciplinary strategies that combine microbiology, oncology, immunology, and systems biology will open new areas of diagnostic and therapeutic innovation for oral cancer.

## 7 Conclusions

The interaction between oral cancer and *Streptococcus* species is highly sophisticated, highlighting the increasing contribution of the microbiome to carcinogenesis. In the present review, we consolidate evidence towards species-specific associations where certain oral streptococci, such as *S. mutans, S. anginosus*, and *S. gordonii* are enriched in oral cancer tissues and contribute to pro-tumorigenic processes through mechanisms that involve chronic inflammation, metabolic reprogramming, and synergistic biofilm formation. On the other hand, the relative abundance of commensal streptococci such as *S. sanguinis, S. mitis*, and *S. oralis* tends to decline as cancer progresses, suggesting a protective, possibly anti-inflammatory role. These disparities reflect the dualistic nature of *Streptococcus* in oral carcinoma and the necessity for species- and strain-level resolution of microbiome studies.

Mechanistic interactions of certain cancer-linked streptococci reveal their role in initiating host inflammatory cascades (e.g., IL-6, CD40, and interferon signaling), biofilm resistance by adhesins (e.g., AgI/II, SpaP, CshA/B, and VisA), and carcinogenic metabolite production including acetaldehyde and kynurenic acid. Some findings also suggest their impact on epithelial markers such as CD44 and ALDH1, with possible effects on stemness and tumor microenvironmental dynamics.

Clinically, there are significant therapeutic and diagnostic implications associated with oral streptococci in the treatment of oral cancer. Streptococcal species may serve as an early microbial biomarker for oral cancer recurrence or progression. From therapeutic approaches involving targeted antibiotics, probiotic repopulation, and anti-adhesin agents to inflammation-modulating strategies, all are aimed at restoring microbial homeostasis and eliminating pro-oncogenic signaling. Despite mounting evidence, some gaps persist. Longitudinal research, strain-level descriptions, host-microbe interaction models, and convergent multi-omics analyses are crucial to establish causative reasoning from the correlative effects of *Streptococcus* spp. in oral carcinogenesis. The convergence of microbiology, oncology, and precision medicine may ultimately enable revolutionary approaches to early detection, risk stratification, and microbiome-directed therapies in oral cancer.

## References

[B1] Abe M. Mori Y. Inaki R. Ohata Y. Abe T. Saijo H. . (2014). A case of odontogenic infection by *Streptococcus constellatus* leading to systemic infection in a Cogan’s syndrome patient. Case Rep. Dent. 2014, 1–4. doi: 10.1155/2014/793174, PMID: 25506439 PMC4258373

[B2] Abiko S. Shimizu Y. Ishikawa M. Inoue M. Nakajima K. Kohya R. . (2023). Effects of activation of an alcohol metabolic gene, cigarette smoking, and alcohol intake on the incidence of metachronous gastric cancer in patients who underwent endoscopic resection for gastric cancer: A multicenter retrospective pilot study. JGH Open 7, 305–310. doi: 10.1002/jgh3.12896, PMID: 37125254 PMC10134755

[B3] Abranches J. Zeng L. Kajfasz J. K. Palmer S. R. Chakraborty B. Wen Z. T. . (2018). Biology of oral streptococci. Microbiol. Spectr. 6. doi: 10.1128/microbiolspec.GPP3-0042-2018, PMID: 30338752 PMC6287261

[B4] Alanazi S. A. S. Alduaiji K. T. A. Shetty B. Alrashedi H. A. Acharya B. L. G. Vellappally S. . (2018). Pathogenic features of Streptococcus mutans isolated from dental prosthesis patients and diagnosed cancer patients with dental prosthesis. Microb. Pathog. 116, 356–361. doi: 10.1016/j.micpath.2018.01.037, PMID: 29407234

[B5] Baek D.-H. Lee S.-H. (2023). Anti-inflammatory efficacy of human-derived *Streptococcus salivarius* on periodontopathogen-induced inflammation. J. Microbiol. Biotechnol. 33, 998–1005. doi: 10.4014/jmb.2302.02002, PMID: 37635315 PMC10468666

[B6] Balkwill F. (2009). Tumour necrosis factor and cancer. Nat. Rev. Cancer 9, 361–371. doi: 10.1038/nrc2628, PMID: 19343034

[B7] Balkwill F. Mantovani A. (2001). Inflammation and cancer: back to Virchow? Lancet 357, 539–545. doi: 10.1016/S0140-6736(00)04046-0, PMID: 11229684

[B8] Banavar G. Ogundijo O. Toma R. Rajagopal S. Lim Y. K. Tang K. . (2021). The salivary metatranscriptome as an accurate diagnostic indicator of oral cancer. NPJ Genomic Med. 6, 105. doi: 10.1038/s41525-021-00257-x, PMID: 34880265 PMC8654845

[B9] Baraniya D. Chitrala K. N. Al-Hebshi N. N. (2022). Global transcriptional response of oral squamous cell carcinoma cell lines to health-associated oral bacteria - an *in vitro* study. J. Oral. Microbiol. 14, 2073866. doi: 10.1080/20002297.2022.2073866, PMID: 35600164 PMC9116255

[B10] Baraniya D. Jain V. Lucarelli R. Tam V. Vanderveer L. Puri S. . (2020). Screening of health-associated oral bacteria for anticancer properties in *vitro*. Front. Cell. Infect. Microbiol. 10. doi: 10.3389/fcimb.2020.575656, PMID: 33123499 PMC7573156

[B11] Begić G. Badovinac I. J. Karleuša L. Kralik K. Cvijanovic Peloza O. Kuiš D. . (2023). Streptococcus salivarius as an Important Factor in Dental Biofilm Homeostasis: Influence on Streptococcus mutans and Aggregatibacter actinomycetemcomitans in Mixed Biofilm. Int. J. Mol. Sci. 24, 7249. doi: 10.3390/ijms24087249, PMID: 37108414 PMC10139097

[B12] Bloch S. Hager-Mair F. F. Andrukhov O. Schäffer C. (2024). Oral streptococci: modulators of health and disease. Front. Cell. Infect. Microbiol. 14. doi: 10.3389/fcimb.2024.1357631, PMID: 38456080 PMC10917908

[B13] Boedtkjer E. Pedersen S. F. (2020). The acidic tumor microenvironment as a driver of cancer. Annu. Rev. Physiol. 82, 103–126. doi: 10.1146/annurev-physiol-021119-034627, PMID: 31730395

[B14] Botelho J. Mascarenhas P. Viana J. Proença L. Orlandi M. Leira Y. . (2022). An umbrella review of the evidence linking oral health and systemic noncommunicable diseases. Nat. Commun. 13, 7614. doi: 10.1038/s41467-022-35337-8, PMID: 36494387 PMC9734115

[B15] Bowen W. H. Burne R. A. Wu H. Koo H. (2018). Oral biofilms: pathogens, matrix, and polymicrobial interactions in microenvironments. Trends Microbiol. 26, 229–242. doi: 10.1016/j.tim.2017.09.008, PMID: 29097091 PMC5834367

[B16] Cappellesso F. Mazzone M. Virga F. (2024). Acid affairs in anti-tumour immunity. Cancer Cell Int. 24, 354. doi: 10.1186/s12935-024-03520-0, PMID: 39465367 PMC11514911

[B17] Caselli E. Fabbri C. D’Accolti M. Soffritti I. Bassi C. Mazzacane S. . (2020). Defining the oral microbiome by whole-genome sequencing and resistome analysis: the complexity of the healthy picture. BMC Microbiol. 20, 120. doi: 10.1186/s12866-020-01801-y, PMID: 32423437 PMC7236360

[B18] Chang C. Geng F. Shi X. Li Y. Zhang X. Zhao X. . (2019). The prevalence rate of periodontal pathogens and its association with oral squamous cell carcinoma. Appl. Microbiol. Biotechnol. 103, 1393–1404. doi: 10.1007/s00253-018-9475-6, PMID: 30470868

[B19] Chattopadhyay I. Verma M. Panda M. (2019). Role of oral microbiome signatures in diagnosis and prognosis of oral cancer. Technol. Cancer Res. Treat. 18, 1533033819867354. doi: 10.1177/1533033819867354, PMID: 31370775 PMC6676258

[B20] Chen C. Zhao S. Karnad A. Freeman J. W. (2018). The biology and role of CD44 in cancer progression: therapeutic implications. J. Hematol. Oncol. 11, 64. doi: 10.1186/s13045-018-0605-5, PMID: 29747682 PMC5946470

[B21] Chocolatewala N. Chaturvedi P. Desale R. (2010). The role of bacteria in oral cancer. Indian J. Med. Paediatr. Oncol. 31, 126–131. doi: 10.4103/0971-5851.76195, PMID: 21584217 PMC3089920

[B22] Chrastek D. Hickman S. Sitaranjan D. Vokshi I. Kakisi O. Kadlec J. . (2020). Streptococcus constellatus causing empyema and sepsis, necessitating early surgical decortication. Case Rep. Infect. Dis. 2020, 1–4. doi: 10.1155/2020/4630809, PMID: 32733717 PMC7376396

[B23] Coletta R. D. Yeudall W. A. Salo T. (2024). Current trends on prevalence, risk factors and prevention of oral cancer. Front. Oral. Heal. 5. doi: 10.3389/froh.2024.1505833, PMID: 39606098 PMC11599248

[B24] Daep C. A. Lamont R. J. Demuth D. R. (2008). Interaction of *Porphyromonas gingivalis* with oral streptococci requires a motif that resembles the eukaryotic nuclear receptor box protein-protein interaction domain. Infect. Immun. 76, 3273–3280. doi: 10.1128/IAI.00366-08, PMID: 18474648 PMC2446731

[B25] Das H. Motghare S. (2021). India as “The oral cancer capital of the world”: the rising burden of oral Malignancies across the nation. Int. J. Sci. Healthc. Res. 6, 99–107. doi: 10.52403/ijshr.20210419

[B26] Dashper S. G. Reynolds E. C. (1996). Lactic acid excretion by Streptococcus mutans. Microbiology 142, 33–39. doi: 10.1099/13500872-142-1-33, PMID: 33657745

[B27] de Freitas Filho S. A. J. Coutinho-Camillo C. M. Oliveira K. K. Bettim B. B. Pinto C. A. L. Kowalski L. P. . (2021). Prognostic implications of ALDH1 and notch1 in different subtypes of oral cancer. J. Oncol. 2021, 1–9. doi: 10.1155/2021/6663720, PMID: 33854547 PMC8020805

[B28] de la Cruz-López K. G. Castro-Muñoz L. J. Reyes-Hernández D. O. García-Carrancá A. Manzo-Merino J. (2019). Lactate in the regulation of tumor microenvironment and therapeutic approaches. Front. Oncol. 9. doi: 10.3389/fonc.2019.01143, PMID: 31737570 PMC6839026

[B29] Deo P. Deshmukh R. (2019). Oral microbiome: Unveiling the fundamentals. J. Oral. Maxillofac. Pathol. 23, 122. doi: 10.4103/jomfp.JOMFP_304_18, PMID: 31110428 PMC6503789

[B30] Dorison L. Béchon N. Martin-Gallausiaux C. Chamorro-Rodriguez S. Vitrenko Y. Ouazahrou R. . (2024). Identification of *Veillonella parvula* and *Streptococcus gordonii* adhesins mediating co-aggregation and its impact on physiology and mixed biofilm structure. MBio 15. e0217124. doi: 10.1128/mbio.02171-24, PMID: 39526776 PMC11633186

[B31] Duguid R. (1985). *In-vitro* acid production by the oral bacterium Streptococcus mutans 10449 in various concentrations of glucose, fructose and sucrose. Arch. Oral. Biol. 30, 319–324. doi: 10.1016/0003-9969(85)90004-4, PMID: 3857902

[B32] Edwards A. M. Grossman T. J. Rudney J. D. (2006). *Fusobacterium nucleatum* Transports Noninvasive *Streptococcus cristatus* into Human Epithelial Cells. Infect. Immun. 74, 654–662. doi: 10.1128/IAI.74.1.654-662.2006, PMID: 16369022 PMC1346643

[B33] Edwards A. M. Grossman T. J. Rudney J. D. (2007). Association of a high-molecular weight arginine-binding protein of *Fusobacterium nucleatum* ATCC 10953 with adhesion to secretory immunoglobulin A and coaggregation with *Streptococcus cristatus*. Oral. Microbiol. Immunol. 22, 217–224. doi: 10.1111/j.1399-302X.2006.00343.x, PMID: 17600532

[B34] Engen S. A. Rørvik G. H. Schreurs O. Blix I. J. Schenck K. (2017). The oral commensal Streptococcus mitis activates the aryl hydrocarbon receptor in human oral epithelial cells. Int. J. Oral. Sci. 9, 145–150. doi: 10.1038/ijos.2017.17, PMID: 28621325 PMC5709542

[B35] Engen S. A. Schreurs O. Petersen F. Blix I. J. S. Bækkevold E. S. Schenck K. (2018). The regulatory role of the oral commensal *Streptococcus mitis* on human monocytes. Scand. J. Immunol. 87, 80–87. doi: 10.1111/sji.12636, PMID: 29194752

[B36] expanded Human Oral Microbiome Database Version 3.1 Natl. Inst. Dent. Craniofacial Res. Available online at: https://homd.org/ (Accessed October 4, 2024).

[B37] Faden H. Mohmand M. (2017). Infections associated with Streptococcus constellatus in children. Pediatr. Infect. Dis. J. 36, 1099–1100. doi: 10.1097/INF.0000000000001646, PMID: 28640003

[B38] Fan X. Peters B. A. Jacobs E. J. Gapstur S. M. Purdue M. P. Freedman N. D. . (2018). Drinking alcohol is associated with variation in the human oral microbiome in a large study of American adults. Microbiome 6, 59. doi: 10.1186/s40168-018-0448-x, PMID: 29685174 PMC5914044

[B39] Farias L. A. B. G. Firmino N. N. Sousa M. M. Lira M. L. Meireles L. N. Stolp Â. M. V. . (2023). Streptococcus constellatus causing concomitant extra and intracranial abscesses complicated with sagittal sinus thrombosis. Rev. Inst. Med. Trop. Sao Paulo 65. doi: 10.1590/s1678-9946202365010, PMID: 36722672 PMC9886223

[B40] Fitzsimonds Z. R. Rodriguez-Hernandez C. J. Bagaitkar J. Lamont R. J. (2020). From beyond the pale to the pale riders: the emerging association of bacteria with oral cancer. J. Dent. Res. 99, 604–612. doi: 10.1177/0022034520907341, PMID: 32091956 PMC7243420

[B41] Furuholm J. Uittamo J. Rautaporras N. Välimaa H. Snäll J. (2023). Streptococcus anginosus: a stealthy villain in deep odontogenic abscesses. Odontology 111, 522–530. doi: 10.1007/s10266-022-00763-z, PMID: 36346473 PMC10020309

[B42] Gangane N. M. Ghongade P. V. Patil B. U. Atram M. (2024). Oral cavity cancer incidence and survival trends: A population-based study. J. Cancer Res. Ther. 20, 1446–1452. doi: 10.4103/jcrt.jcrt_2720_22, PMID: 38261454

[B43] Gao Z. Chen X. Wang C. Song J. Xu J. Liu X. . (2024). New strategies and mechanisms for targeting Streptococcus mutans biofilm formation to prevent dental caries: A review. Microbiol. Res. 278, 127526. doi: 10.1016/j.micres.2023.127526, PMID: 39491258

[B44] Gao L. Xu T. Huang G. Jiang S. Gu Y. Chen F. (2018). Oral microbiomes: more and more importance in oral cavity and whole body. Protein Cell 9, 488–500. doi: 10.1007/s13238-018-0548-1, PMID: 29736705 PMC5960472

[B45] Gelfo V. Romaniello D. Mazzeschi M. Sgarzi M. Grilli G. Morselli A. . (2020). Roles of IL-1 in cancer: from tumor progression to resistance to targeted therapies. Int. J. Mol. Sci. 21, 6009. doi: 10.3390/ijms21176009, PMID: 32825489 PMC7503335

[B46] Gevers D. Knight R. Petrosino J. F. Huang K. McGuire A. L. Birren B. W. . (2012). The human microbiome project: A community resource for the healthy human microbiome. PloS Biol. 10, e1001377. doi: 10.1371/journal.pbio.1001377, PMID: 22904687 PMC3419203

[B47] Glover J. Kovacevic G. Walton G. Parr D. (2020). Fulminating deep tissue space infection with Streptococcus constellatus presenting initially as a sore throat. BMJ Case Rep. 13, e233971. doi: 10.1136/bcr-2019-233971, PMID: 32234857 PMC7167435

[B48] Guo L. Shokeen B. He X. Shi W. Lux R. (2017). *Streptococcus mutans* SpaP binds to RadD of *Fusobacterium nucleatum* ssp. *polymorphum*. Mol. Oral. Microbiol. 32, 355–364. doi: 10.1111/omi.12177, PMID: 27976528 PMC5472499

[B49] Haruta I. Kikuchi K. Hashimoto E. Kato H. Hirota K. Kobayashi M. . (2008). A possible role of histone-like DNA-binding protein of Streptococcus intermedius in the pathogenesis of bile duct damage in primary biliary cirrhosis. Clin. Immunol. 127, 245–251. doi: 10.1016/j.clim.2008.01.010, PMID: 18337173

[B50] Hayes R. B. Ahn J. Fan X. Peters B. A. Ma Y. Yang L. . (2018). Association of oral microbiome with risk for incident head and neck squamous cell cancer. JAMA Oncol. 4, 358. doi: 10.1001/jamaoncol.2017.4777, PMID: 29327043 PMC5885828

[B51] He X. Hu W. Kaplan C. W. Guo L. Shi W. Lux R. (2012). Adherence to Streptococci Facilitates Fusobacterium nucleatum Integration into an Oral Microbial Community. Microb. Ecol. 63, 532–542. doi: 10.1007/s00248-011-9989-2, PMID: 22202886 PMC3313671

[B52] Hung T.-Y. Phuong L. K. Grobler A. Tong S. Y. C. Freeth P. Pelenda A. . (2024). Antibiotics to eradicate Streptococcus pyogenes pharyngeal carriage in asymptomatic children and adults: A systematic review. J. Infect. 88, 106104. doi: 10.1016/j.jinf.2024.01.003, PMID: 38360357

[B53] Inui I. Mochizuki S. Hirabayashi-Nishimuta F. Yoshioka Y. Takahashi O. Sasaguri M. . (2025). *In vitro* impact of Streptococcus mitis on the inhibition of oral cancer cell proliferation *via* mitotic modulation. Front. Cell. Infect. Microbiol. 15. doi: 10.3389/fcimb.2025.1524820, PMID: 40415961 PMC12098639

[B54] Issa E. Salloum T. Tokajian S. (2020). From normal flora to brain abscesses: A review of streptococcus intermedius. Front. Microbiol. 11. doi: 10.3389/fmicb.2020.00826, PMID: 32457718 PMC7221147

[B55] Ito S. Misaki T. Naka S. Wato K. Nagasawa Y. Nomura R. . (2019). Specific strains of Streptococcus mutans, a pathogen of dental caries, in the tonsils, are associated with IgA nephropathy. Sci. Rep. 9, 20130. doi: 10.1038/s41598-019-56679-2, PMID: 31882880 PMC6934739

[B56] Jiang S. Li M. Fu T. Shan F. Jiang L. Shao Z. (2020). Clinical characteristics of infections caused by Streptococcus anginosus group. Sci. Rep. 10, 9032. doi: 10.1038/s41598-020-65977-z, PMID: 32493976 PMC7270121

[B57] Kaci G. Goudercourt D. Dennin V. Pot B. Doré J. Ehrlich S. D. . (2014). Anti-inflammatory properties of streptococcus salivarius, a commensal bacterium of the oral cavity and digestive tract. Appl. Environ. Microbiol. 80, 928–934. doi: 10.1128/AEM.03133-13, PMID: 24271166 PMC3911234

[B58] Karpiński T. M. (2019). Role of oral microbiota in cancer development. Microorganisms 7, 20. doi: 10.3390/microorganisms7010020, PMID: 30642137 PMC6352272

[B59] Kilian M. (2018). The oral microbiome – friend or foe? Eur. J. Oral. Sci. 126, 5–12. doi: 10.1111/eos.12527, PMID: 30178561

[B60] Kleinstein S. E. Nelson K. E. Freire M. (2020). Inflammatory networks linking oral microbiome with systemic health and disease. J. Dent. Res. 99, 1131–1139. doi: 10.1177/0022034520926126, PMID: 32459164 PMC7443998

[B61] Kobo O. Nikola S. Geffen Y. Paul M. (2017). The pyogenic potential of the different Streptococcus anginosus group bacterial species: retrospective cohort study. Epidemiol. Infect. 145, 3065–3069. doi: 10.1017/S0950268817001807, PMID: 28803566 PMC9152745

[B62] Kojima A. Nakano K. Wada K. Takahashi H. Katayama K. Yoneda M. . (2012). Infection of specific strains of Streptococcus mutans, oral bacteria, confers a risk of ulcerative colitis. Sci. Rep. 2, 332. doi: 10.1038/srep00332, PMID: 22451861 PMC3312205

[B63] Kolenbrander P. E. Palmer R. J. Periasamy S. Jakubovics N. S. (2010). Oral multispecies biofilm development and the key role of cell–cell distance. Nat. Rev. Microbiol. 8, 471–480. doi: 10.1038/nrmicro2381, PMID: 20514044

[B64] Korona-Glowniak I. Skawinska-Bednarczyk A. Wrobel R. Pietrak J. Tkacz-Ciebiera I. Maslanko-Switala M. . (2022). Streptococcus sobrinus as a Predominant Oral Bacteria Related to the Occurrence of Dental Caries in Polish Children at 12 Years Old. Int. J. Environ. Res. Public Health 19, 15005. doi: 10.3390/ijerph192215005, PMID: 36429724 PMC9690266

[B65] Krzyściak W. Jurczak A. Kościelniak D. Bystrowska B. Skalniak A. (2014). The virulence of Streptococcus mutans and the ability to form biofilms. Eur. J. Clin. Microbiol. Infect. Dis. 33, 499–515. doi: 10.1007/s10096-013-1993-7, PMID: 24154653 PMC3953549

[B66] Kurkivuori J. Salaspuro V. Kaihovaara P. Kari K. Rautemaa R. Grönroos L. . (2007). Acetaldehyde production from ethanol by oral streptococci. Oral. Oncol. 43, 181–186. doi: 10.1016/j.oraloncology.2006.02.005, PMID: 16859955

[B67] Kwak S. Wang C. Usyk M. Wu F. Freedman N. D. Huang W.-Y. . (2024). Oral microbiome and subsequent risk of head and neck squamous cell cancer. JAMA Oncol. 10, 1537. doi: 10.1001/jamaoncol.2024.4006, PMID: 39325441 PMC11428028

[B68] Lamont R. J. Koo H. Hajishengallis G. (2018). The oral microbiota: dynamic communities and host interactions. Nat. Rev. Microbiol. 16, 745–759. doi: 10.1038/s41579-018-0089-x, PMID: 30301974 PMC6278837

[B69] La Rosa G. Gattuso G. Pedulla E. Rapisarda E. Nicolosi D. Salmeri M. (2020). Association of oral dysbiosis with oral cancer development (Review). Oncol. Lett. 19 (4), 3045–3058. doi: 10.3892/ol.2020.11441, PMID: 32211076 PMC7079586

[B70] Latifi-Xhemajli B. Rexhepi A. Veronneau J. Kutllovci T. Ahmeti D. Bajrami S. (2021). Streptococcus mutans infections in infants and related maternal and child factors. Acta Stomatol. Croat. 55, 308–315. doi: 10.15644/asc55/3/8, PMID: 34658377 PMC8514224

[B71] Lekshmi Priya K. S. Maheswary D. Ravi S. S. S. Leela K. V. Lathakumari R. H. Malavika G. (2025). The impact of probiotics on oral cancer: Mechanistic insights and therapeutic strategies. Oral. Oncol. Rep. 13, 100715. doi: 10.1016/j.oor.2025.100715

[B72] Lemos J. A. Palmer S. R. Zeng L. Wen Z. T. Kajfasz J. K. Freires I. A. . (2019). The biology of Streptococcus mutans. Microbiol. Spectr. 7. doi: 10.1128/microbiolspec.GPP3-0051-2018, PMID: 30657107 PMC6615571

[B73] Li W. Liang H. Lin X. Hu T. Wu Z. He W. . (2023a). A catalog of bacterial reference genomes from cultivated human oral bacteria. NPJ Biofilms Microbiomes 9, 45. doi: 10.1038/s41522-023-00414-3, PMID: 37400465 PMC10318035

[B74] Li Y. Saraithong P. Zhang L. Dills A. Paster B. J. Xiao J. . (2023b). Dynamics of oral microbiome acquisition in healthy infants: A pilot study. Front. Oral. Heal. 4. doi: 10.3389/froh.2023.1152601, PMID: 37065420 PMC10098328

[B75] Liao Y. Zhang J.-B. Lu L.-X. Jia Y.-J. Zheng M.-Q. Debelius J. W. . (2023). Oral microbiota alteration and roles in epstein-barr virus reactivation in nasopharyngeal carcinoma. Microbiol. Spectr. 11. doi: 10.1128/spectrum.03448-22, PMID: 36645283 PMC9927204

[B76] Liberti M. V. Locasale J. W. (2016). The Warburg effect: how does it benefit cancer cells? Trends Biochem. Sci. 41, 211–218. doi: 10.1016/j.tibs.2015.12.001, PMID: 26778478 PMC4783224

[B77] Lima B. P. Shi W. Lux R. (2017). Identification and characterization of a novel *Fusobacterium nucleatum* adhesin involved in physical interaction and biofilm formation with *Streptococcus gordonii*. Microbiologyopen 6, e00444. doi: 10.1002/mbo3.444, PMID: 28173636 PMC5458471

[B78] Lin K.-Y. Chung C.-H. Ciou J.-S. Su P.-F. Wang P.-W. Shieh D.-B. . (2019). Molecular damage and responses of oral keratinocyte to hydrogen peroxide. BMC Oral. Health 19, 10. doi: 10.1186/s12903-018-0694-0, PMID: 30634966 PMC6329095

[B79] Liu G. Qiao Y. Zhang Y. Leng C. Chen H. Sun J. . (2020). Metabolic Profiles of Carbohydrates in Streptococcus thermophilus During pH-Controlled Batch Fermentation. Front. Microbiol. 11. doi: 10.3389/fmicb.2020.01131, PMID: 32547529 PMC7272703

[B80] López-Lázaro M. (2007). Dual role of hydrogen peroxide in cancer: Possible relevance to cancer chemoprevention and therapy. Cancer Lett. 252, 1–8. doi: 10.1016/j.canlet.2006.10.029, PMID: 17150302

[B81] Lu Z. Zhang C. Zhang J. Su W. Wang G. Wang Z. (2025). The kynurenine pathway and indole pathway in tryptophan metabolism influence tumor progression. Cancer Med. 14, e70703. doi: 10.1002/cam4.70703, PMID: 40103267 PMC11919716

[B82] Mäkinen A. I. Pappalardo V. Y. Buijs M. J. Brandt B. W. Mäkitie A. A. Meurman J. H. . (2023). Salivary microbiome profiles of oral cancer patients analyzed before and after treatment. Microbiome 11, 171. doi: 10.1186/s40168-023-01613-y, PMID: 37542310 PMC10403937

[B83] Matsui R. Cvitkovitch D. (2010). Acid tolerance mechanisms utilized by *Streptococcus mutans*. Future Microbiol. 5, 403–417. doi: 10.2217/fmb.09.129, PMID: 20210551 PMC2937171

[B84] Mattos-Graner R. O. Klein M. I. Smith D. J. (2014). Lessons learned from clinical studies: roles of mutans streptococci in the pathogenesis of dental caries. Curr. Oral. Heal. Rep. 1, 70–78. doi: 10.1007/s40496-013-0008-1

[B85] Meroni G. Panelli S. Zuccotti G. Bandi C. Drago L. Pistone D. (2021). Probiotics as therapeutic tools against pathogenic biofilms: have we found the perfect weapon? Microbiol. Res. (Pavia) 12, 916–937. doi: 10.3390/microbiolres12040068

[B86] Milane L. S. (2022). “ The hallmarks of cancer and immunology,” in Cancer Immunology and Immunotherapy (United Kingdom: Elsevier), 1–17. doi: 10.1016/B978-0-12-823397-9.00001-6

[B87] Mok S. F. Karuthan C. Cheah Y. K. Ngeow W. C. Rosnah Z. Yap S. F. . (2017). The oral microbiome community variations associated with normal, potentially Malignant disorders and Malignant lesions of the oral cavity. Malays. J. Pathol. 39, 1–15., PMID: 28413200

[B88] Morita E. Narikiyo M. Yano A. Nishimura E. Igaki H. Sasaki H. . (2003). Different frequencies of Streptococcus anginosus infection in oral cancer and esophageal cancer. Cancer Sci. 94, 492–496. doi: 10.1111/j.1349-7006.2003.tb01471.x, PMID: 12824872 PMC11160302

[B89] National Institute of Dental and Craniofacial Research (2025). Oral Cancer 5-Year Survival Rates by Race, Sex, and Stage of Diagnosis ( NIDCR Web Page). Available online at: https://www.nidcr.nih.gov/research/data-statistics/oral-cancer/survival-rates (Accessed April 2, 2025).

[B90] Neumayr A. Kubitz R. Bode J. Bilk P. Häussinger D. (2010). Multiple liver abscesses with isolation of streptococcus intermedius related to a pyogenic dental infection in an immuno-competent patient. Eur. J. Med. Res. 15, 319. doi: 10.1186/2047-783X-15-7-319, PMID: 20696645 PMC3351958

[B91] Nobbs A. H. Lamont R. J. Jenkinson H. F. (2009). *Streptococcus* adherence and colonization. Microbiol. Mol. Biol. Rev. 73, 407–450. doi: 10.1128/MMBR.00014-09, PMID: 19721085 PMC2738137

[B92] Nomura R. Matayoshi S. Otsugu M. Kitamura T. Teramoto N. Nakano K. (2020). Contribution of severe dental caries induced by streptococcus mutans to the pathogenicity of infective endocarditis. Infect. Immun. 88. doi: 10.1128/IAI.00897-19, PMID: 32312765 PMC7309618

[B93] Nosova T. Jousimies-Somer H. Kaihovaara P. Jokelainen K. Heine R. Salaspuro M. (1997). Characteristics of alcohol dehydrogenases of certain aerobic bacteria representing human colonic flora. Alcohol. Clin. Exp. Res. 21, 489–494. doi: 10.1111/j.1530-0277.1997.tb03795.x 9161610

[B94] Nuriel-Ohayon M. Neuman H. Koren O. (2016). Microbial changes during pregnancy, birth, and infancy. Front. Microbiol. 7. doi: 10.3389/fmicb.2016.01031, PMID: 27471494 PMC4943946

[B95] Ohshima J. Wang Q. Fitzsimonds Z. R. Miller D. P. Sztukowska M. N. Jung Y.-J. . (2019). *Streptococcus gordonii* programs epithelial cells to resist ZEB2 induction by *Porphyromonas gingivalis*. Proc. Natl. Acad. Sci. 116, 8544–8553. doi: 10.1073/pnas.1900101116, PMID: 30971493 PMC6486779

[B96] Okahashi N. Nakata M. Kuwata H. Kawabata S. (2022). Oral mitis group streptococci: A silent majority in our oral cavity. Microbiol. Immunol. 66, 539–551. doi: 10.1111/1348-0421.13028, PMID: 36114681

[B97] Okahashi N. Nakata M. Sumitomo T. Terao Y. Kawabata S. (2013). Hydrogen peroxide produced by oral streptococci induces macrophage cell death. PloS One 8, e62563. doi: 10.1371/journal.pone.0062563, PMID: 23658745 PMC3643943

[B98] Park J.-A. Lee G. R. Lee J.-Y. Jin B.-H. (2023). Oral probiotics, streptococcus salivarius K12 and M18, suppress the release of volatile sulfur compounds and a virulent protease from oral bacteria: an *in-vitro* study. Oral. Health Prev. Dent. 21, 259–270. doi: 10.3290/j.ohpd.b4328987, PMID: 37724895 PMC11619835

[B99] Peres M. A. Macpherson L. M. D. Weyant R. J. Daly B. Venturelli R. Mathur M. R. . (2019). Oral diseases: a global public health challenge. Lancet 394, 249–260. doi: 10.1016/S0140-6736(19)31146-8, PMID: 31327369

[B100] Pilarczyk-Zurek M. Sitkiewicz I. Koziel J. (2022). The clinical view on streptococcus anginosus group – opportunistic pathogens coming out of hiding. Front. Microbiol. 13. doi: 10.3389/fmicb.2022.956677, PMID: 35898914 PMC9309248

[B101] Pimenta F. Gertz R. E. Park S. H. Kim E. Moura I. Milucky J. . (2019). Streptococcus infantis, Streptococcus mitis, and Streptococcus oralis Strains With Highly Similar cps5 Loci and Antigenic Relatedness to Serotype 5 Pneumococci. Front. Microbiol. 9. doi: 10.3389/fmicb.2018.03199, PMID: 30671034 PMC6332807

[B102] Pushalkar S. Ji X. Li Y. Estilo C. Yegnanarayana R. Singh B. . (2012). Comparison of oral microbiota in tumor and non-tumor tissues of patients with oral squamous cell carcinoma. BMC Microbiol. 12, 144. doi: 10.1186/1471-2180-12-144, PMID: 22817758 PMC3507910

[B103] Rams T. E. Feik D. Mortensen J. E. Degener J. E. van Winkelhoff A. J. (2014). Antibiotic Susceptibility of Periodontal Streptococcus constellatus and Streptococcus intermedius Clinical Isolates. J. Periodontol. 85, 1792–1798. doi: 10.1902/jop.2014.130291, PMID: 25102269

[B104] Rašková M. Lacina L. Kejík Z. Venhauerová A. Skaličková M. Kolář M. . (2022). The role of IL-6 in cancer cell invasiveness and metastasis—Overview and therapeutic opportunities. Cells 11, 3698. doi: 10.3390/cells11223698, PMID: 36429126 PMC9688109

[B105] Rébé C. Ghiringhelli F. (2020). Interleukin-1β and cancer. Cancers (Basel) 12, 1791. doi: 10.3390/cancers12071791, PMID: 32635472 PMC7408158

[B106] Ren J. Sun P. Wang M. Zhou W. Liu Z. (2024). Insights into the role of Streptococcus oralis as an opportunistic pathogen in infectious diseases. Front. Cell. Infect. Microbiol. 14. doi: 10.3389/fcimb.2024.1480961, PMID: 39559706 PMC11570589

[B107] Ren J.-Y. Yu H.-Q. Xu S. Zhou W.-J. Liu Z.-H. (2025). Putative pathogenic factors underlying Streptococcus oralis opportunistic infections. J. Microbiol. Immunol. Infect. 58, 157–163. doi: 10.1016/j.jmii.2024.09.001, PMID: 39261123

[B108] Reyes J. V. M. Dondapati M. Ahmad S. Song D. Lieber J. J. Pokhrel N. B. . (2023). A case report of multiple abscesses caused by *Streptococcus intermedius*. Clin. Case Rep. 11, e6813. doi: 10.1002/ccr3.6813, PMID: 36694650 PMC9842781

[B109] Rickard A. H. Gilbert P. High N. J. Kolenbrander P. E. Handley P. S. (2003). Bacterial coaggregation: an integral process in the development of multi-species biofilms. Trends Microbiol. 11, 94–100. doi: 10.1016/S0966-842X(02)00034-3, PMID: 12598132

[B110] Rostami N. Shields R. C. Serrage H. J. Lawler C. Brittan J. L. Yassin S. . (2022). Interspecies competition in oral biofilms mediated by Streptococcus gordonii extracellular deoxyribonuclease SsnA. NPJ Biofilms Microbiomes 8, 96. doi: 10.1038/s41522-022-00359-z, PMID: 36509765 PMC9744736

[B111] Ryntathiang I. Babu N. A. Selvan S. T. Dharmalingam Jothinathan M. K. (2024). Impact of lifestyle factors on oral cancer risk and prevention: Oral cancer epidemiology. Oral. Oncol. Rep. 9, 100259. doi: 10.1016/j.oor.2024.100259

[B112] Şenel S. (2021). An overview of physical, microbiological and immune barriers of oral mucosa. Int. J. Mol. Sci. 22, 7821. doi: 10.3390/ijms22157821, PMID: 34360589 PMC8346143

[B113] Sakamoto H. Naito H. Ohta Y. Tanakna R. Maeda N. Sasaki J. . (1999a). Isolation of bacteria from cervical lymph nodes in patients with oral cancer. Arch. Oral. Biol. 44, 789–793. doi: 10.1016/S0003-9969(99)00079-5, PMID: 10530911

[B114] Sakamoto H. Sasaki J. Nord C. E. (1999b). Association between bacterial colonization on the tumor, bacterial translocation to the cervical lymph nodes and subsequent postoperative infection in patients with oral cancer. Clin. Microbiol. Infect. 5, 612–616. doi: 10.1111/j.1469-0691.1999.tb00417.x, PMID: 11851691

[B115] Sasaki M. Yamaura C. Ohara-Nemoto Y. Tajika S. Kodama Y. Ohya T. . (2005). Streptococcus anginosus infection in oral cancer and its infection route. Oral. Dis. 11, 151–156. doi: 10.1111/j.1601-0825.2005.01051.x, PMID: 15888105

[B116] Saxena P. S. Kumar P. S. (2019). Non-habit related oral squamous cell carcinoma: possible etiologic factors and probable prevention in Indian scenario. Oral. Surg. Oral. Med. Oral. Pathol. Oral. Radiol. 128, e90. doi: 10.1016/j.oooo.2019.02.231

[B117] Schultze L. B. Maldonado A. Lussi A. Sculean A. Eick S. (2021). The impact of the pH value on biofilm formation. Oral Biofilms (Karger) 29, 19–29. doi: 10.1159/000510196, PMID: 33427214

[B118] Sedghi L. DiMassa V. Harrington A. Lynch S. V. Kapila Y. L. (2021). The oral microbiome: Role of key organisms and complex networks in oral health and disease. Periodontol. 2000 87, 107–131. doi: 10.1111/prd.12393, PMID: 34463991 PMC8457218

[B119] Seitz H. K. Stickel F. (2010). Acetaldehyde as an underestimated risk factor for cancer development: role of genetics in ethanol metabolism. Genes Nutr. 5, 121–128. doi: 10.1007/s12263-009-0154-1, PMID: 19847467 PMC2885165

[B120] Senthil Kumar S. Gunda V. Reinartz D. M. Pond K. W. Thorne C. A. Santiago Raj P. V. . (2024a). Oral streptococci S. anginosus and S. mitis induce distinct morphological, inflammatory, and metabolic signatures in macrophages. Infect. Immun. 92. doi: 10.1128/iai.00536-23, PMID: 38289109 PMC10929413

[B121] Senthil Kumar S. Johnson M. D. L. Wilson J. E. (2024b). Insights into the enigma of oral streptococci in carcinogenesis. Microbiol. Mol. Biol. Rev. 88. doi: 10.1128/mmbr.00095-23, PMID: 38506551 PMC11338076

[B122] Shehata S. A. Toraih E. A. Ismail E. A. Hagras A. M. Elmorsy E. Fawzy M. S. (2023). Vaping, environmental toxicants exposure, and lung cancer risk. Cancers (Basel) 15, 4525. doi: 10.3390/cancers15184525, PMID: 37760496 PMC10526315

[B123] Smith D. J. Anderson J. M. King W. F. van Houte J. Taubman M. A. (1993). Oral streptococcal colonization of infants. Oral. Microbiol. Immunol. 8, 1–4. doi: 10.1111/j.1399-302X.1993.tb00535.x, PMID: 8510978

[B124] Socransky S. S. Haffajee A. D. Cugini M. A. Smith C. Kent R. L. (1998). Microbial complexes in subgingival plaque. J. Clin. Periodontol. 25, 134–144. doi: 10.1111/j.1600-051X.1998.tb02419.x, PMID: 9495612

[B125] Spatafora G. Li Y. He X. Cowan A. Tanner A. C. R. (2024). The evolving microbiome of dental caries. Microorganisms 12, 121. doi: 10.3390/microorganisms12010121, PMID: 38257948 PMC10819217

[B126] Sudhakara P. Gupta A. Bhardwaj A. Wilson A. (2018). Oral dysbiotic communities and their implications in systemic diseases. Dent. J. 6, 10. doi: 10.3390/dj6020010, PMID: 29659479 PMC6023521

[B127] Sukmana B. I. Saleh R. O. Najim M. A. AL-Ghamdi H. S. Achmad H. Al-Hamdani M. M. . (2024). Oral microbiota and oral squamous cell carcinoma: a review of their relation and carcinogenic mechanisms. Front. Oncol. 14. doi: 10.3389/fonc.2024.1319777, PMID: 38375155 PMC10876296

[B128] Tackett A. P. Urman R. Barrington-Trimis J. Liu F. Hong H. Pentz M. A. . (2024). Prospective study of e-cigarette use and respiratory symptoms in adolescents and young adults. Thorax 79, 163–168. doi: 10.1136/thorax-2022-218670, PMID: 37582630 PMC11062480

[B129] Tan Y. Wang Z. Xu M. Li B. Huang Z. Qin S. . (2023). Oral squamous cell carcinomas: state of the field and emerging directions. Int. J. Oral. Sci. 15, 44. doi: 10.1038/s41368-023-00249-w, PMID: 37736748 PMC10517027

[B130] Tang Y. L. Sim T. S. Tan K. S. (2022). Oral streptococci subvert the host innate immune response through hydrogen peroxide. Sci. Rep. 12, 656. doi: 10.1038/s41598-021-04562-4, PMID: 35027607 PMC8758666

[B131] Torralba M. G. Aleti G. Li W. Moncera K. J. Lin Y.-H. Yu Y. . (2021). Oral microbial species and virulence factors associated with oral squamous cell carcinoma. Microb. Ecol. 82, 1030–1046. doi: 10.1007/s00248-020-01596-5, PMID: 33155101 PMC8551143

[B132] Tran M. P. Caldwell-McMillan M. Khalife W. Young V. B. (2008). Streptococcus intermedius causing infective endocarditis and abscesses: a report of three cases and review of the literature. BMC Infect. Dis. 8, 154. doi: 10.1186/1471-2334-8-154, PMID: 18992173 PMC2600825

[B133] Tsai M.-S. Chen Y.-Y. Chen W.-C. Chen M.-F. (2022). Streptococcus mutans promotes tumor progression in oral squamous cell carcinoma. J. Cancer 13, 3358–3367. doi: 10.7150/jca.73310, PMID: 36186905 PMC9516012

[B134] Tuominen H. Collado M. C. Rautava J. Syrjänen S. Rautava S. (2019). Composition and maternal origin of the neonatal oral cavity microbiota. J. Oral. Microbiol. 11, 1663084. doi: 10.1080/20002297.2019.1663084, PMID: 31528268 PMC6735328

[B135] Väkeväinen S. Tillonen J. Salaspuro M. (2001). 4-methylpyrazole decreases salivary acetaldehyde levels in ALDH2-deficient subjects but not in subjects with normal ALDH2. Alcohol. Clin. Exp. Res. 25, 829–834. doi: 10.1111/j.1530-0277.2001.tb02286.x, PMID: 11410717

[B136] Vesty A. Gear K. Boutell S. Taylor M. W. Douglas R. G. Biswas K. (2020). Randomised, double-blind, placebo-controlled trial of oral probiotic Streptococcus salivarius M18 on head and neck cancer patients post-radiotherapy: a pilot study. Sci. Rep. 10, 13201. doi: 10.1038/s41598-020-70024-y, PMID: 32764634 PMC7411050

[B137] Vulisha A. K. Sam R. Nur H. Bhardwaj N. Sirineni S. (2021). Aggressive presentation of streptococcus constellatus. Cureus. 13 (4), e14534. doi: 10.7759/cureus.14534, PMID: 34017651 PMC8128152

[B138] Wang Y. Wu J. Lv M. Shao Z. Hungwe M. Wang J. . (2021). Metabolism characteristics of lactic acid bacteria and the expanding applications in food industry. Front. Bioeng. Biotechnol. 9. doi: 10.3389/fbioe.2021.612285, PMID: 34055755 PMC8149962

[B139] Wei Y. Li Y. Chen Y. Liu P. Huang S. Zhang Y. . (2022). ALDH1: A potential therapeutic target for cancer stem cells in solid tumors. Front. Oncol. 12. doi: 10.3389/fonc.2022.1026278, PMID: 36387165 PMC9650078

[B140] World Health Organization (2022). Global Oral Health Status Report Towards Universal Health Coverage for Oral Health by 2030. Executive Summary. (Geneva: World Health Organization).

[B141] Wu S. Cao Z. Lu R. Zhang Z. Sethi G. You Y. (2025). Interleukin-6 (IL-6)-associated tumor microenvironment remodelling and cancer immunotherapy. Cytokine Growth Factor Rev. 85, 93–102. doi: 10.1016/j.cytogfr.2025.01.001, PMID: 39828476

[B142] Xia J. Xia L. Zhou H. Lin X. Xu F. (2021). Empyema caused by Streptococcus constellatus: a case report and literature review. BMC Infect. Dis. 21, 1267. doi: 10.1186/s12879-021-06955-2, PMID: 34930151 PMC8686261

[B143] Xiao H. Li Y. (2025). From teeth to body: the complex role of *Streptococcus mutans* in systemic diseases. Mol. Oral. Microbiol. 40, 65–81. doi: 10.1111/omi.12491, PMID: 39865888

[B144] Xu W. Bradstreet T. R. Zou Z. Hickerson S. Zhou Y. He H. . (2025). Reprogramming aerobic metabolism mitigates Streptococcus pyogenes tissue damage in a mouse necrotizing skin infection model. Nat. Commun. 16, 2559. doi: 10.1038/s41467-025-57348-x, PMID: 40089471 PMC11910614

[B145] Xu Y. Jia Y. Chen L. Gao J. Yang D. (2021). Effect of Streptococcus anginosus on biological response of tongue squamous cell carcinoma cells. BMC Oral. Health 21, 141. doi: 10.1186/s12903-021-01505-3, PMID: 33743656 PMC7981962

[B146] Yang R. Liu T. Pang C. Cai Y. Lin Z. Guo L. . (2022). The Regulatory Effect of Coaggregation Between Fusobacterium nucleatum and Streptococcus gordonii on the Synergistic Virulence to Human Gingival Epithelial Cells. Front. Cell. Infect. Microbiol. 12. doi: 10.3389/fcimb.2022.879423, PMID: 35573793 PMC9100429

[B147] Yang C. Washio J. Lin Y. Hsu M. Wang D. Tsai F. . (2025). Microbiome signatures and dysbiotic patterns in oral cancer and precancerous lesions. Oral. Dis. 31 (8), 2456–2465. doi: 10.1111/odi.15317, PMID: 40106821 PMC12423479

[B148] Yang C.-Y. Yeh Y.-M. Yu H.-Y. Chin C.-Y. Hsu C.-W. Liu H. . (2018). Oral microbiota community dynamics associated with oral squamous cell carcinoma staging. Front. Microbiol. 9. doi: 10.3389/fmicb.2018.00862, PMID: 29774014 PMC5943489

[B149] Yu L. Hong Y. Maishi N. Matsuda A. Y. Hida Y. Hasebe A. . (2024). Oral bacterium *Streptococcus mutans* promotes tumor metastasis through thrombosis formation. Cancer Sci. 115, 648–659. doi: 10.1111/cas.16010, PMID: 38096871 PMC10859626

[B150] Yu L. Maishi N. Akahori E. Hasebe A. Takeda R. Matsuda A. Y. . (2022). The oral bacterium Streptococcus mutans promotes tumor metastasis by inducing vascular inflammation. Cancer Sci. 113, 3980–3994. doi: 10.1111/cas.15538, PMID: 35997541 PMC9633306

[B151] Zhang Y. Fang J. Yang J. Gao X. Dong L. Zheng X. . (2022). Streptococcus mutans - associated bacteria in dental plaque of severe early childhood caries. J. Oral. Microbiol. 14, 2046309. doi: 10.1080/20002297.2022.2046309, PMID: 35251525 PMC8896182

[B152] Zhang X. Hyer J. M. Yu H. D’Silva N. J. Kirkwood K. L. (2014). DUSP1 phosphatase regulates the proinflammatory milieu in head and neck squamous cell carcinoma. Cancer Res. 74, 7191–7197. doi: 10.1158/0008-5472.CAN-14-1379, PMID: 25312268 PMC4268021

[B153] Zhao H. Chu M. Huang Z. Yang X. Ran S. Hu B. . (2017). Variations in oral microbiota associated with oral cancer. Sci. Rep. 7, 11773. doi: 10.1038/s41598-017-11779-9, PMID: 28924229 PMC5603520

[B154] Zheng L. Itzek A. Chen Z. Kreth J. (2011). Environmental influences on competitive hydrogen peroxide production in streptococcus gordonii. Appl. Environ. Microbiol. 77, 4318–4328. doi: 10.1128/AEM.00309-11, PMID: 21571883 PMC3127700

[B155] Zhou J. Hu Z. Wang L. Hu Q. Chen Z. Lin T. . (2024). Tumor-colonized Streptococcus mutans metabolically reprograms tumor microenvironment and promotes oral squamous cell carcinoma. Microbiome 12, 193. doi: 10.1186/s40168-024-01907-9, PMID: 39369210 PMC11452938

[B156] Zhu X. Chen J. Wu S. Zeng J. Sun Y. Wu X. (2024). Empyema Caused by Mixed Infection with Streptococcus intermedius and Streptococcus constellatus in a Patient with Previous Surgery for Oral Carcinoma: A Case Report. Infect. Drug Resist. 17, 4447–4454. doi: 10.2147/IDR.S490700, PMID: 39431214 PMC11491076

[B157] Zhu Y.-J. Li S.-Y. Yang S.-S. Du Y. Zhang Z.-Y. Liu J.-Y. (2025). CD44 on cancer stem cell is a potential immunological and prognostic pan-cancer biomarker. Cancer Cell Int. 25, 134. doi: 10.1186/s12935-025-03748-4, PMID: 40200220 PMC11978154

[B158] Zu Y. Li W. Wang Q. Chen J. Guo Q. (2019). ComDE two-component signal transduction systems in oral streptococci: structure and function. Curr. Issues Mol. Biol. 32, 201–258. doi: 10.21775/cimb.032.201, PMID: 31166173

